# Emergence of a Persistent Surface-Normal Dynamical Mode in Planar Polymer Brushes

**DOI:** 10.1021/acsmacrolett.6c00437

**Published:** 2026-09-18

**Authors:** Ankit Saha, Michael J. A. Hore

**Affiliations:** Department of Macromolecular Science and Engineering, 2546 Case Western Reserve University, 10900 Euclid Avenue, Cleveland, Ohio 44106, United States

## Abstract

Polymer grafting affects chain relaxation dynamics in ways that can strongly influence the physical properties of polymer nanocomposites and surface layers. Here, we performed energy-conserving dissipative particle dynamics (eDPD) simulations with proper orthogonal decomposition (POD) to investigate the dominant relaxation modes of planar polymer brushes. While the dynamics parallel to the substrate remain largely consistent with classical Rouse behavior, the perpendicular dynamics collapse into a single persistent, surface-localized mode that remains remarkably robust across polymer chain lengths and grafting densities. We show that this mode can be represented by a localized exponential contribution together with a small number of low-order Rouse modes. While the spatial extent of the localized contribution increases with grafting density, the local strength of the graft-induced perturbation becomes approximately independent of grafting density once a brush is formed. Further analysis of the contour-resolved dynamical memory confirms that grafting produces increasingly anisotropic dynamics that extend farther along the polymer contour with increasing grafting density. These results provide a mechanistic framework for understanding how grafting reorganizes polymer dynamics and identify a surface-localized dynamical mode that may underlie the suppression of long-wavelength Rouse modes observed in neutron scattering experiments.

## Graphical abstract

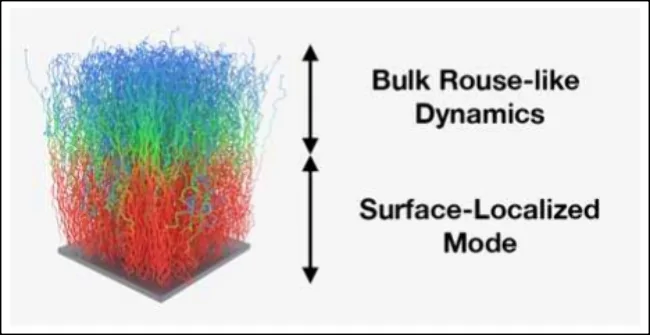

Polymer chain dynamics strongly influence the mechanical, transport, and rheological properties of polymeric materials. For example, recent work by Koga and co-workers^1,2^ combined neutron spectroscopy with molecular dynamics simulations to determine how the topological loops formed by adsorbed polymers on nanofiller surfaces dictate the mechanical response of materials similar to those used in car tires. They observed that SiO_2_ and carbon black (CB) fillers reinforced polybutadiene (PBD) through distinct topological loop structures formed by polymer chains adsorbed on the filler surfaces. The densely packed, relatively small loops formed on silane-treated SiO_2_ suppressed interdigitation with the surrounding matrix, whereas the larger, more widely spaced loops on CB promoted interdigitation and stronger filler–polymer connectivity. Analysis of the neutron scattering data further revealed a suppression of long-wavelength Rouse modes^3^ associated with the adsorbed polymer chains. Earlier neutron spin echo spectroscopy (NSE) measurements by Richter et al.^4^ observed a similar phenomenon in polymer melts during the transition from unentangled to entangled behavior, with long wavelength modes suppressed due to the spatial confinement of entangled chains. Although these studies demonstrate that confinement suppresses long-wavelength chain motions, they do not directly reveal how grafting modifies the underlying patterns of collective polymer motion.

Grafting polymers to surfaces profoundly modifies both their equilibrium conformations and their relaxation dynamics through excluded-volume interactions and geometric constraints.^5–13^ In addition to controlling the toughness of the materials they comprise,^14^ the combination of polymer conformation and altered relaxation dynamics also influences transport properties^15–19^ and the glass transition temperature.^20^ On spherical nanoparticle surfaces at moderately high grafting densities σ, two regions of conformation develop with segments of the chains aligned perpendicular to the surface close to the nanoparticle core, which eventually transition to more ideal conformations farther away.^12,21–23^ As a result, segments of the grafted chains located in the inner region of the brush, which experience stronger confinement, relax 2–3 times more slowly than those farther away.^24^ This behavior has been attributed to the curvature of the nanoparticle surface.^10,12,13^ For sufficiently large nanoparticles, where surface curvature becomes negligible, the dynamics are expected to approach those of planar brushes. Grafting polymers to surfaces may also introduce entirely new collective motions. For example, NSE measurements of planar brushes confirmed de Gennes’ prediction of the emergence of a “breathing mode”, consisting of correlated monomer displacements perpendicular to the surface that has no analog for free chains.^25^ For an idealized polymer constrained at one end, modification of the Rouse boundary conditions gives rise to half-integer (odd) modes rather than the integer cosine spectrum of a free chain.^26–29^ Recent NSE measurements by Kruteva et al. confirmed our earlier DPD finding that grafted-chain dynamics cannot be adequately described by the classical Rouse model.^10,30^ Their analysis further revealed strong contour-position dependence and dynamical heterogeneity, with an enhanced monomeric friction localized near the grafting point. Despite these advances, whether grafting fundamentally changes the intrinsic dynamical eigenmodes of individual polymer chains, rather than simply modifying their relaxation times, remains largely unexplored.

Previous work has demonstrated that proper orthogonal decomposition (POD)^31^ quantitatively reproduces the Rouse eigenmodes and relaxation spectra of free polymer chains directly from molecular simulation trajectories.^10,32–34^ Here we show that such analyses can provide more than a compact description of motion, and can reveal the physical mechanisms responsible for the observed dynamics. Data-driven techniques like POD infer structure, patterns, or governing relationships directly from data, with minimal reliance on *a priori* model assumptions. In this Letter, we use POD to extract physical insight by solving the inverse problem: identifying the minimal modification to the Rouse model required to reproduce modes obtained directly from dissipative particle dynamics (DPD) simulations. We focus on a planar brush in contact with a good solvent. While relaxation modes parallel to the substrate remain unchanged from those of a free chain, the perpendicular dynamics collapse into a single hybridized mode that mixes multiple low-order Rouse modes with a spatially localized, exponentially decaying contribution. We demonstrate that this mode arises from a boundary-induced perturbation to the Rouse operator with a strength that is insensitive to changes in chain length or grafting density, but which extends farther along the polymer contour with increasing grafting density. These results provide a mechanistic explanation of how boundary-induced perturbations produced by grafting reshape the modal content of the polymers, and, consequently, lead to changes in their relaxation dynamics.

We performed energy-conserving dissipative particle dynamics (eDPD) simulations of planar brushes in solution at a temperature below the critical temperature (*T*_C_ = 1.0 ϵ_0_/*k*_B_) for different polymer chain lengths (*N*) and grafting densities (σ). eDPD has been shown to be an effective simulation framework for studying thermoresponsive polymers,^35,36^ and its use here was motivated by a desire to connect the behavior of this system to our previous work that focused on free chains.^34^ The full details of our methods are contained in the Supporting Information. We kept the temperature fixed at *T* = 0.8 ϵ_0_/*k*_B_ which produced good solvent conditions for the brush. After equilibration, defined as the time at which the mean-squared end-to-end distance saturated to a constant value, 10^6^ production time steps were performed with monomer coordinates stored every 500 time steps for postsimulation analysis by POD. Shown in Figure 1 are representative snapshots of grafted polymer systems for chain lengths of *N* = 20, 50, and 100 (rows, top to bottom) at grafting densities of σ = 0.25, 0.50, 0.75, 1.00, 1.25, and 1.50 *r*_c_^–2^ (columns, left to right). *r*_c_ ≈ 1 nm is the unit of length in DPD, defined as the cutoff distance of the main interaction force. The range of grafting densities was selected to represent a range capturing common experimental choices for both planar brushes and polymer-grafted nanoparticles.^37^ Representative chains are highlighted in blue to illustrate individual chain conformations. As σ increases, the chains appear more elongated and aligned in the direction perpendicular to the substrate as a result of increased crowding. We quantified the brush height by the root mean-squared end-to-end distance of the grafted polymers and verified that the brush height *H* ∼ *N* ^ν^, where ν increases from 0.95 (σ = 0.25 *r*_c_^–2^) to 1.0 (σ ≥ 0.50 *r*_c_^–2^) indicating the formation of brushes for the systems we considered (Table S9, Supporting Information).

**Figure 1:**
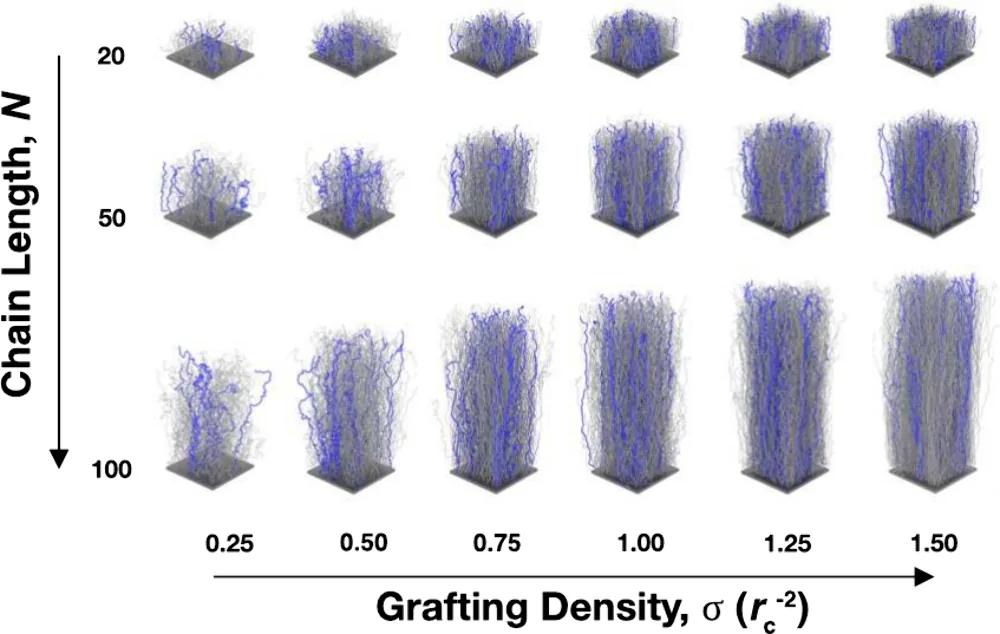
Representative snapshots of planar brushes formed from chains of length *N* = 20, 50, and 100 (top to bottom), and grafting densities from σ = 0.25 *r*_c_^–2^ to 1.50 *r*_c_^–2^ (left to right). Selected polymers are shaded blue to highlight their individual conformations. The snapshots have been corrected for the periodic boundary condition using the minimum image convention.

Previous work^10,32–34^ has demonstrated that the dynamical modes extracted by POD for free polymer chains quantitatively agree with the modes predicted from the Rouse model,

$$\phi_{p}\left(s\right)\approx \sqrt{\frac{2}{N}}\cos\left(\pi ps\right)$$ 1

with associated relaxation times that also scale with *N* as predicted (e.g., τ_1_ ∼ *N* ^2^ in the absence of hydrodynamics). The normalized contour index *s* = *n*/*N* and varies between 0 (grafted end) and 1 (free end). As the mode number *p* increases, the modes describe shorter wavelength motions along the chain contour. Shown in Figure 2A are the *p* = 1 modes in the direction parallel to the substrate normalized by the value of the mode at the grafted chain end, ψ_∥_(*s*)/ψ_∥_(0), for the three values of *N* we considered. For comparison, the *p* = 1 normalized Rouse mode, ϕ_1_(*s*)/ϕ_1_(0), is plotted as a dashed line. Note that although our previous studies^10,34^ have found quantitative agreement between the POD modes and the Rouse modes, it is not necessarily the case that they are the same. POD simply extracts the dominant motions of the system from the coordinate snapshots of the monomers.

**Figure 2:**
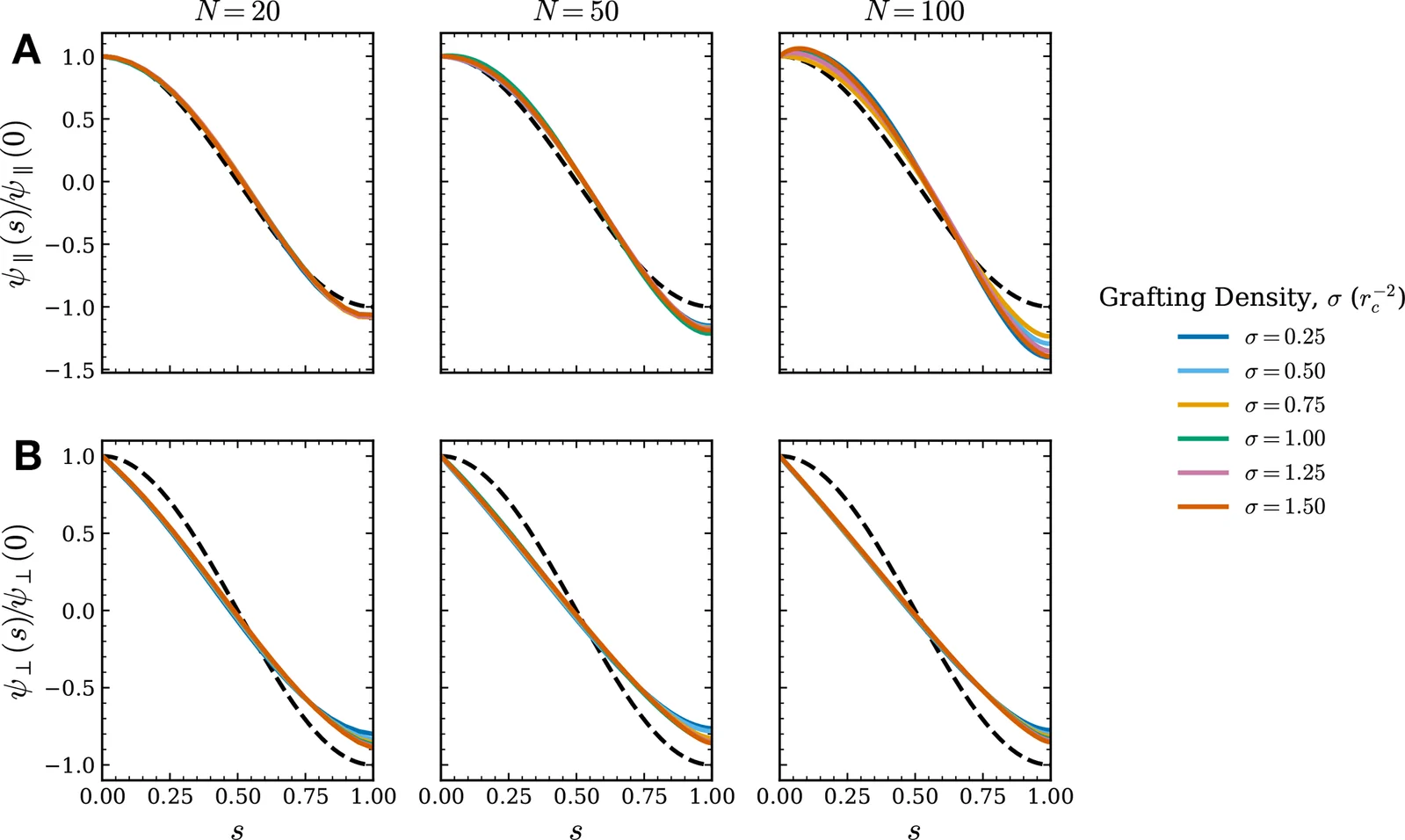
Normalized mode amplitudes for *p* = 1 in the (A) parallel and (B) perpendicular directions, plotted as a function of the normalized contour index *s* = *n*/*N*. *s* = 0 is the grafted chain end. Columns from left to right correspond to *N* = 20, 50, and 100, respectively. Colors correspond to different grafting densities as indicated in the legend. The dashed black line is the lowest order Rouse mode ϕ_1_(*s*), shown for comparison.

The leading POD mode accounts for approximately 70% of the total variance of the parallel dynamics, with the remaining variance distributed primarily among the next few modes. As *N* increases, the agreement between the Rouse mode and POD mode decreases for large values of *s*, and the POD mode is no longer antisymmetric about *s* = 1/2. In all cases, |ψ_∥_(0)| < |ψ_∥_(1)| indicating that, within the dominant parallel displacement pattern, the free end undergoes larger displacements than the grafted end. This effect becomes more amplified as *N* increases. We attribute this behavior to two sources. First, grafting one chain end to the substrate restricts the motion of that monomer and perturbs the motions of those near it. This can be observed by the immediate increase in the magnitude of the mode in the vicinity of *s* = 0 as *s* increases. Second, the free chain end is less confined than portions of the chain deep within the brush and is able to move more freely. Although one might expect a systematic variation in the value of ψ_∥_(1) with respect to σ we do not observe one. This nonmonotonic behavior may reflect conformational heterogeneity near the brush periphery. Nevertheless, the overall shape of the parallel mode remains quite similar to the Rouse mode, indicating that the fundamental displacement patterns in the parallel direction are not significantly perturbed from those of a free chain.

In contrast, perpendicular to the substrate we find only a single, highly perturbed mode is present (Figure 2B) which accounts for essentially all of the variance in the monomer displacements in this direction. The mode is significantly different from the first Rouse mode (dashed line), and unlike in the parallel direction, maintains a similar shape for all chain lengths and grafting densities. In addition, the perpendicular mode shape remains reproducible across multiple independent simulations suggesting that it is a robust consequence of the grafting condition on the polymers. As a limiting case, we also performed simulations of an isolated grafted chain. A characteristic surface-normal mode persists in the absence of neighboring chains (Figures S7–S9), demonstrating that its emergence does not require interchain crowding. The grafting constraint itself appears sufficient to establish the basic contour-space structure of the mode, while interchain interactions within the brush modify its quantitative characteristics.

Numerous studies have sought to explain deviations from ideal Rouse dynamics arising from confinement, heterogeneous friction, and intermolecular interactions.^9,12,13,28,30,38^ The classical sinusoidal Rouse modes arise as the eigenfunctions of the Rouse operator (with appropriate boundary conditions), defined through the eigenvalue problem:

$$K\phi_{p}=\lambda_{p}\phi_{p}$$ 2

where the unperturbed Rouse operator **K** represents the discrete connectivity (Laplacian) operator generated by the harmonic springs between neighboring monomers. Because the observed mode remains localized near the grafting surface across all chain lengths and grafting densities, we hypothesized that grafting acts as a localized perturbation of the Rouse operator, such that **K** → **K** + δ**K**. Motivated by this perturbative picture, we represent the perturbed eigenmode as the sum of a surface-localized exponential contribution and a finite superposition of Rouse modes. Notably, these terms arise without needing to specify the exact form of δ**K**, and we obtain an expression for the perpendicular POD mode:

$$\psi_{⊥}\left(s\right)=A_{\exp}\left[e^{-s/\xi }-{\left\langle e^{-s/\xi }\right\rangle }_{s}\right]+\overset{p_{\max}}{\underset{p=1}{\sum }}A_{p}\phi_{p}\left(s\right)$$ 3

The first term represents a surface-localized contribution to ψ_⊥_(*s*), and *A*_exp_ and *A*_*p*_ denote the amplitudes of the surface-localized term and Rouse modes, respectively. ⟨···⟩_*s*_ denotes the average over the contour, and ϕ_*p*_(*s*) are the usual Rouse cosine modes. We found that only the first few lowest order Rouse modes contributed to ψ_⊥_(*s*) and fixed *p*_max_ = 5. Because the center-of-mass motion was removed before performing POD, every internal POD mode must have zero contour average, and the contour average of the exponential is subtracted so that it remains orthogonal to the translational mode. A full derivation is provided in the Supporting Information. Fits to this function are shown in the Supporting Information, Section 4 (Figures S1–S6) for both the parallel and perpendicular directions, with a summary of values presented in Section 4.2 (Tables S1–S6). In the parallel direction, *A*_exp_ ≈ 0 and *A*_*p*_ ≈ 0 for *p* > 2, suggesting the lowest-order mode is essentially the lowest-order Rouse mode with a small contribution from the *p* = 2 mode. The contribution from the higher-order modes increases as *N* increases, but remains relatively constant with respect to σ. Perpendicular to the substrate, we find a significant contribution from the surface-localized term and a Rouse-like contribution that comes almost exclusively from the *p* = 1 Rouse mode. The amplitude of the Rouse component *A*_1_ decreases as σ increases, while *A*_exp_ increases as σ increases indicating that the surface-localized component becomes amplified as the grafting density (i.e., confinement of the chains) increases. The amplitudes of the higher-order modes are small, and remain relatively constant with respect to σ. Finally, as σ increases, ξ also increases implying that the distortion of the mode from the grafting condition extends farther along the chain contour as σ increases. A representative fit to ψ_⊥_(*s*) for *N* = 100 and σ = 1.5 *r*_c_^–2^ is shown in Figure 3A, showing the contributions of the surface-localized term and the Rouse modes. For this mode, ξ = 0.819 indicating that the surface-localized contribution decays over a characteristic length spanning approximately 82% of the chain contour.

**Figure 3:**
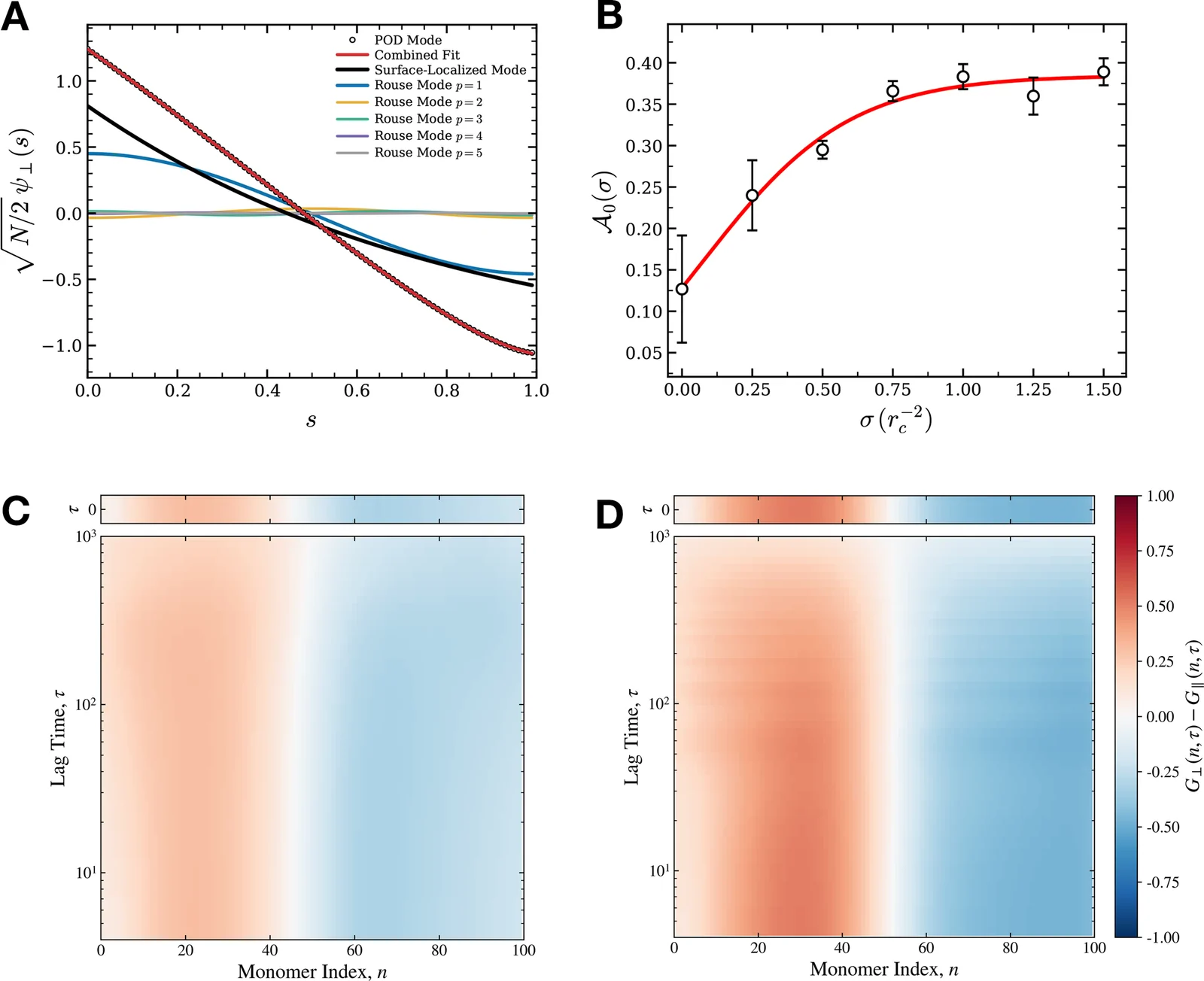
(A) Representative perpendicular mode for *N* = 100 and σ = 1.50 *r*_c_^–2^, along with a least-squares fit to our model in eq 3. The black line is the surface-localized term, the blue line is ϕ_1_(*s*), and the remaining lines are contributions from higher order Rouse modes (2 ≤ *p* ≤ 5). (B) Contour-averaged dynamical anisotropy $A_{0}$ as a function of grafting density σ. Error bars represent one standard deviation in $A_{0}$ . The red line is a visual guide. (C, D) Anisotropic contour-resolved dynamical memory corresponding to σ = 0.25 and 1.50 *r*_c_^–2^, respectively, demonstrating the increase in directional dynamical anisotropy with increasing grafting density.

To gain physical insight into the behavior of the brush, we examined the contour-resolved dynamical memory of the polymer chains. We computed the normalized time-lagged memory

$$G_{\alpha }\left(n,\tau \right)=\frac{1}{k}\overset{k-1}{\underset{m=0}{\sum }}\frac{\left\langle \delta r_{\alpha }\left(n,t+\tau \right)\delta r_{\alpha }\left(m,t\right)\right\rangle }{\sqrt{\left\langle \delta r_{\alpha }{\left(n,t\right)}^{2}\right\rangle \left\langle \delta r_{\alpha }{\left(m,t\right)}^{2}\right\rangle }}$$ 4

where *k* = 5 defines a small reference region comprising the five monomers nearest the graft point, and α = *x*, *y* or *z*. Here, δ*r*_α_(*n*, *t*) denotes the fluctuation of monomer *n* about its time-averaged position in the instantaneous chain center-of-mass reference frame. *G*_α_(*n*, τ) measures how strongly the motion of a monomer at contour position *n* remains dynamically linked to the motion of the graft-proximal reference region after a time delay τ in direction α. A large positive value of *G*_α_(*n*, τ) signifies that the two regions tend to move in the same direction after τ, whereas negative values indicate persistent anticorrelated motion. The magnitude of *G*_α_(*n*, τ) reflects the strength of this dynamical memory. We examined *G*_∥_(*n*, τ) = [*G*_*x*_(*n*, τ) + *G*_*y*_(*n*, τ)]/2 and *G*_⊥_(*n*, τ) = *G*_*z*_(*n*, τ), and computed the contour-averaged dynamical anisotropy for each grafting density:

$$A_{0}\left(\sigma \right)={\left\{\frac{1}{N}\underset{n}{\sum }{\left[G_{⊥}\left(n,0\right)-G_{∥}\left(n,0\right)\right]}^{2}\right\}}^{1/2}$$ 5

which quantifies the contour-averaged difference between the perpendicular and parallel dynamical memory at zero lag time.

Shown in Figure 3B is $A_{0}\left(\sigma \right)$ for *N* = 100. As the grafting density increases, $A_{0}$ increases before approaching a plateau for σ ≥ 0.75 *r*_*c*_^–2^, indicating that confinement progressively strengthens the directional anisotropy of the collective brush dynamics before saturating. Analogous plots for *N* = 20 and *N* = 50 are shown in the Supporting Information (Figures S10 and S11). Although the general behavior of $A_{0}$ with respect to σ is similar for different *N*, we found a small chain-length dependence that slightly increases $A_{0}$ as *N* increases. Importantly, this increasing dynamical anisotropy is not inconsistent with the comparatively weak dependence of the POD mode shapes on grafting density observed in Figure 2. The POD eigenvectors ψ­(*s*) describe the contour-space structure of the correlated motion, whereas $A_{0}$ quantifies the difference between the perpendicular and parallel dynamical memory. Thus, increasing grafting density can strengthen the anisotropy of the dynamics without substantially altering the spatial structure of the dominant modes. Increasing polymer grafting density therefore leads to more strongly confined chains and greater anisotropy between the perpendicular and parallel dynamics, while the characteristic contour-space structure of these motions remains comparatively insensitive to σ.

We sought to better understand the behavior of $A_{0}$ through the normalized contour-resolved dynamical memory of the polymer chains, as shown in Figure 3C (σ = 0.25 *r*_*c*_^–2^) and D (σ = 1.50 *r*_c_^–2^). In these heat maps, positive values indicate that dynamical memory persists more strongly in the perpendicular direction, whereas negative values indicate relatively stronger memory parallel to the substrate. For τ = 0, both heat maps exhibit an increase in the dynamical memory from *n* = 0 to approximately *n* = 30, followed by a gradual decrease toward the free end. This implies that if the region near the graft point fluctuates, the first few monomers remain strongly constrained by the graft and exhibit comparatively little independent motion. As *n* increases toward *n* ≈ 30, however, the chain gains conformational freedom and this region can move more collectively with the region near the grafting point. Beyond the maximum near *n* ≈ 30, the remaining portion of the chain becomes progressively less constrained by the graft, and the chain begins behaving more independently. This behavior becomes substantially more pronounced as the grafting density increases, as evidenced by the larger positive values throughout Figure 3D. Interestingly, although the leading edge of the anisotropic region shifts toward larger contour positions with increasing σ, the position of the maximum near *n* ≈ 30 changes comparatively little. Thus, increasing grafting density more strongly affects the magnitude and spatial extent of the anisotropic dynamics than the contour position of their maximum. This weak shift does not imply that the spatial extent of the surface perturbation is independent of σ. The reduced-basis analysis shows that ξ generally increases with grafting density. Rather, the features in *G*(*n*, τ) reflect the full chain dynamics, including both the surface-localized contribution and the underlying chain modes, and therefore need not scale directly with ξ. The hot spot formed in the heat maps in the vicinity of *n* ≈ 30 is observed to persist over a considerable amount of time. The persistence of this feature to τ > 10^2^ τ_0_ motivates our description of the surface-normal mode as persistent. Although the overall spatial pattern of the anisotropy remains nearly unchanged with increasing lag time, its magnitude steadily decreases. This observation suggests that the localized dynamical structure established by the grafting constraint persists over time but progressively loses coherence as the chain relaxes.

To quantify the growth of the surface-localized dynamical region with increasing σ, we examined the properties of the exponential contribution to ψ_⊥_(*s*). The Rouse modes satisfy the condition that ∂ϕ_*p*_/∂*s* = 0 at *s* = 0. For the POD mode, the surface gradient ∂ψ_⊥_/∂*s* = −*A*_exp_/ξ = −Γ_⊥_ at *s* = 0. This value quantifies the magnitude of the graft-induced distortion at the substrate. Shown in Figure 4A are the values of Γ_⊥_ as a function of σ for all three grafted chain lengths. Note that the larger uncertainties for *N* = 20 likely arise from the coarse contour resolution of the surface-localized region, which is represented by only a few monomers and therefore limits the precision with which Γ_⊥_ can be determined. Nevertheless, Γ_⊥_ displays the same qualitative behavior with respect to σ for all values of *N* investigated. The single-chain simulations, shown as asterisks in Figure 4A, provide a low-density reference and exhibit larger uncertainties than the brush simulations. Within these uncertainties, the results are consistent with a decrease in Γ_⊥_ from the single-chain regime before reaching a relatively stable value of Γ_⊥_ ≈ 2.25 for σ > 0.50 *r*_c_^–2^. This implies that the local surface gradient becomes relatively insensitive to either σ or *N* once a sufficiently dense brush is formed. Within this brush regime, the approximate stability of Γ_⊥_ results from the simultaneous variation of *A*_exp_ and ξ with grafting density. Thus, while the local strength of the perturbation becomes approximately independent of σ, its spatial extent continues to increase, as demonstrated by the increase in ξ. The relative Rouse contribution simultaneously decreases. To verify that Γ_⊥_ accurately captures the local surface gradient, we independently estimated the gradient directly from the first few contour points of the measured ψ_⊥_ mode (Γ_⊥_^0^). As shown in Figure 4B, the ratio Γ_⊥_^0^/Γ_⊥_ remains close to unity within uncertainty across the grafting densities and chain lengths investigated, demonstrating that the exponential contribution correctly reproduces the local behavior of the perpendicular mode near the graft.

**Figure 4:**
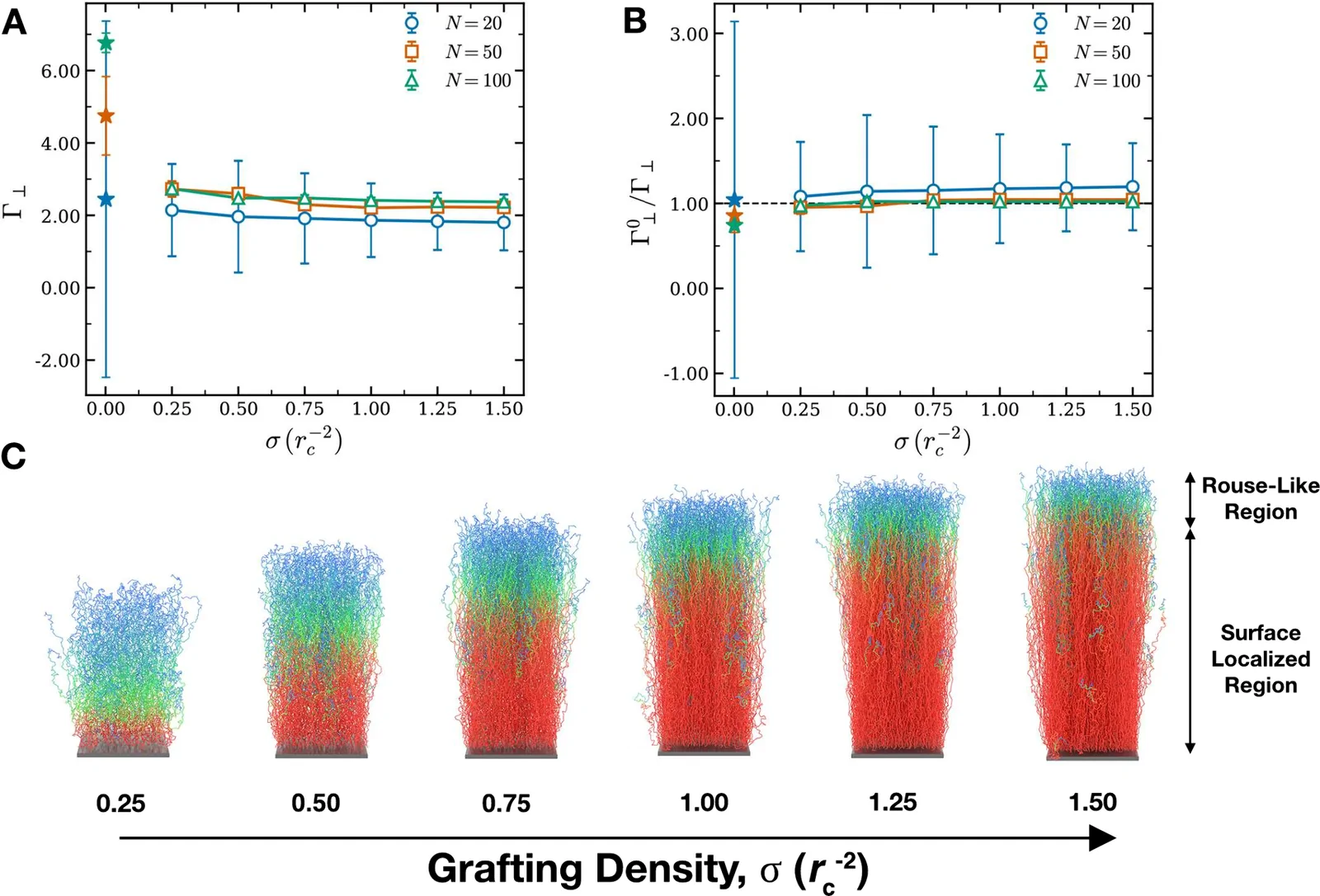
(A) Surface gradient Γ_⊥_ = *A*_exp_/ξ plotted as a function of grafting density for *N* = 20 (blue circles), *N* = 50 (orange squares), and *N* = 100 (green triangles). (B) Directly measured surface gradient Γ_⊥_^0^ normalized by the fitted surface gradient values shown in (A). Asterisks denote isolated grafted-chain simulations (σ = 0.0017 *r*_c_^–2^) and are shown as low-density controls rather than as part of the connected brush series. (C) Representative snapshots of brushes for *N* = 100. The red region indicates one characteristic decay length, *N*ξ, of the surface-localized contribution. It should not be interpreted as a sharp boundary. The green-to-blue color gradient is a visual aid indicating progression along the chain contour from the grafting surface toward the free chain end.

The increase in ξ with σ can be visualized in Figure 4C, where representative snapshots of the brush for *N* = 100 have been rendered and shaded according to *N*ξ. *N*ξ represents a characteristic decay length of the surface-localized contribution, with *n* < *N*ξ shaded as red, and the remainder of the chain in green/blue. The green-to-blue color gradient is a visual aid indicating progression along the chain contour from the grafting surface toward the free chain end. It does not encode an additional physical quantity or classification based on ξ. The evolution of *N*ξ closely mirrors the increase of $A_{0}$ , supporting the conclusion that the growing dynamical anisotropy originates from an increasing extent of the graft-induced perturbation along the contour rather than an increase in its strength.

Collectively, these results demonstrate that grafting fundamentally reorganizes polymer relaxation by generating a robust, surface-localized dynamical mode that mixes with a smaller remnant of the classical Rouse spectrum. By combining data-driven discovery with a simple perturbative interpretation of the Rouse model, we provide a framework for understanding how the grafting condition reshapes the dynamics of the polymer brush. Notably, our simulations show that the dynamical consequence of grafting polymers to a planar substrate is not a uniform modification of the entire relaxation spectrum, but the emergence of a single surface-localized mode in the perpendicular direction. One implication of these results is that the surface-localized mode may provide a microscopic mechanism contributing to the suppression of long-wavelength Rouse modes observed by NSE in confined polymers.

Although we have focused exclusively on a brush in contact with a good solvent, how solvent quality – including θ-solvent conditions and polymer melts – influences planar brush dynamics remains an open question of particular importance for nanocomposite applications. We are currently investigating this problem, and preliminary simulations indicate that the surface-normal mode persists under different solvent conditions. Whether its dependence on grafting density and chain length remains unchanged, however, is not yet established; quantities such as ξ, *A*_exp_, and $A_{0}$ may vary with solvent quality. These preliminary observations motivate our ongoing investigation of which features of the surface-normal mode are universal consequences of grafting and which depend on the surrounding polymer environment. Extending this framework to different solvent conditions, entangled brushes, curved interfaces, adsorbed chains, and polymer-grafted nanoparticles should ultimately establish whether surface-localized modal perturbations provide a general description of grafted-polymer dynamics. Additionally, how the presence or absence of free chain ends, as well as their distribution throughout the brush, influences dynamical anisotropy remains an intriguing open question. For example, constraining the free end through loop formation would provide a direct means of examining how chain-end freedom contributes to the distinction between surface-parallel and surface-normal dynamics. More broadly, this work illustrates how data-driven analysis can move beyond describing polymer dynamics to identifying the physical mechanisms that generate them, providing a framework for studying complex polymer relaxation phenomena that lack tractable analytical solutions.

## Supplementary Material



## Notes

Details of the energy-conserving dissipative particle dynamics (eDPD) simulations, implementation of proper orthogonal decomposition (POD), derivation of the discrete eigenmode analysis for the perturbed Rouse operator, reduced-basis representation and fitting of the POD modes, fitted mode parameters for all chain lengths and grafting densities, brush-height scaling data, and additional dynamical anisotropy results (PDF)
