# Supplementary material for: Emergence of a Persistent Surface-Normal Dynamical Mode in Planar Polymer Brushes
Source: ACS Macro Lett. 2026 Sep 18;15(10):1550–7. doi: 10.1021/acsmacrolett.6c00437 (PMC13689921; doi:10.1021/acsmacrolett.6c00437)
Supplement: Supplementary file 1 [file mz6c00437_si_001.pdf]

Supporting Information:
Emergence of a Persistent Surface-Normal Dynamical Mode in Planar Polymer Brushes

Ankit Saha[1] and Michael J. A. Hore[1,*]

[1]Department of Macromolecular Science and Engineering, Case Western Reserve University, Cleveland, OH 44106, United States.
*E-mail: hore@case.edu

Contents

1 Energy-Conserving Dissipative Particle Dynamics (eDPD) S2

2 Proper Orthogonal Decomposition (POD) S3

3 Discrete Eigenmode Analysis of a Perturbed Rouse Operator S4
3.1 Eigenmodes of the Unperturbed Discrete Rouse Operator . . . S4
3.2 Surface-Localized Solution of a Perturbed Operator . . . S5
3.3 Reduced Basis Representation of the POD Modes . . . S6

4 Polymer Brush Simulation Results S8
4.1 Fitting Results . . . S8
4.1.1 Parallel Direction . . . S8
4.1.2 Perpendicular Direction . . . S11
4.2 Summary of Reduced Basis Fitting Parameters . . . S14
4.2.1 $N = 20$ . . . S14
4.2.2 $N = 50$ . . . S15
4.2.3 $N = 100$ . . . S16

5 Single Chain Control Simulation Results S17
5.1 Fitting Results . . . S17
5.2 Summary of Reduced Basis Fitting Parameters . . . S18

6 Brush Heights, H S19

7 Dynamical Anisotropy S20

1 Energy-Conserving Dissipative Particle Dynamics (eDPD)

Energy-conserving dissipative particle dynamics (eDPD) simulations were performed on planar, grafted polymer systems to explore the role of chain length (N), grafting density (σ), and favorable/unfavorable interactions on chain dynamics. eDPD is a coarse-grained technique that groups several monomers or solvent molecules into a single fluid element (i.e., DPD beads). In eDPD, the system is kept in contact with a thermal reservoir with a temperature T_r. Each DPD bead i is assigned a temperature T_i, and the system temperature $\langle T \rangle = n_b^{-1} \sum_i T_i$. As described below, we control $\langle T \rangle$ by setting T_r and allowing the system to reach thermal equilibrium. We kept $T_r = 0.8\ \epsilon_0/k_B$ fixed for this study. Simulations were performed in fixed boxes with dimensions of $24 \times 24 \times 96\ r_c^3$ ($L_x \times L_y \times L_z$), with periodic boundary conditions in all three directions. $r_c = 1.0$ is the fundamental length scale in DPD. A substrate with thickness $h = 2\ r_c$ was aligned parallel to the xy-plane at the bottom of the simulation box.[1] The substrate was created by arranging DPD beads into a rigid face-centered cubic lattice (lattice constant $l = 0.22\ r_c$) and was kept fixed for the duration of the simulations.[1] We varied the grafting density of polymer from $\sigma = 0.25\ r_c^{-2}$ to $1.50\ r_c^{-2}$ in increments of $0.25\ r_c^{-2}$. The solvent density was $\rho = 3\ r_c^{-3}$. The number of DPD beads $n_b = \sigma N L_x L_y + \rho L_x L_y (L_z - h)$.

The implementation we employ in this work closely resembles that used in a prior study by Hore.[2] A total of n_b beads were simulated, and beads i and j interact through three forces when their separation distance $r_{ij} = |\mathbf{r}_{ij}| < r_c$. Previous studies have estimated that $r_c \approx 1$ nm for commonly-used parameters (e.g., ζ_{ij}, ρ, etc.)[3,4] The three primary forces consist of a conservative force $\mathbf{F}_{ij}^C$ that represents soft-repulsive interactions between particles, as well as a random force ($\mathbf{F}_{ij}^R$) and dissipative force ($\mathbf{F}_{ij}^D$) which together form the thermostat of the simulations. These forces are defined by:

$$\mathbf{F}_{ij}^C = a_{ij} w^R(r_{ij}) \hat{\mathbf{r}}_{ij}, \tag{1}$$

$$\mathbf{F}_{ij}^R = \zeta_{ij} (\Delta t)^{-1/2} w^R(r_{ij}) \theta_{ij} \hat{\mathbf{r}}_{ij}, \tag{2}$$

and

$$\mathbf{F}_{ij}^D = -\gamma_{ij} w^D(r_{ij}) (\hat{\mathbf{r}}_{ij} \cdot \mathbf{v}_{ij}) \hat{\mathbf{r}}_{ij}, \tag{3}$$

where $\mathbf{r}_{ij} = \mathbf{r}_i - \mathbf{r}_j$, $\hat{\mathbf{r}}_{ij} = \mathbf{r}_{ij}/r_{ij}$, and $\mathbf{v}_{ij} = \mathbf{v}_i - \mathbf{v}_j$. The variables ζ_{ij} and γ_{ij} are related to the strength of thermal fluctuations and energy dissipation respectively. In the case of eDPD, the fluctuation-dissipation theorem requires that the random coefficient is related to the dissipative coefficient and the temperatures of the interacting particles (T_i and T_j): $\zeta_{ij} = 4\gamma_{ij} k_B T_i T_j/(T_i + T_j)$. This means that unlike traditional DPD, the values of ζ_{ij} and γ_{ij} must be calculated at every time step for each pair of interacting particles. w^D and w^R are weighting factors for the dissipative and random forces, respectively. They are related to each other as: $w^D(r_{ij}) = [w^R(r_{ij})]^2$, with $w^R(r_{ij}) = (1 - r_{ij}/r_c)$ if $r < r_c$ and 0 otherwise.

The coefficient a_{ij} represents the strength of the conservative interaction between particles i and j, and this value becomes larger as the interaction grows more unfavorable. Our system has a critical temperature $T_C = 1.0\ \epsilon_0/k_B$, and for monomer–solvent interactions, a_{ij} varies with temperature as:

$$a_{ij}(T) = \Lambda_0 + \frac{75\epsilon_0}{\rho r_c^4} + \frac{\Delta\Lambda}{1 + \exp[\mu(T - T_C)]} \tag{4}$$

with the coefficients $\Lambda_0 = -10\ \epsilon_0/r_c$, $\Delta\Lambda = 20\ \epsilon_0/r_c$, and $\mu = 300\ k_B/\epsilon_0$. ϵ_0 is the fundamental energy unit in DPD, with $\epsilon_0 = 1.0$. The parameters in Eq. 4 were taken from previous eDPD studies of LCST polymers, and produce a sharp increase in the interaction strength at $T = T_C$.[2,5,6] Monomer–monomer and solvent–solvent interactions did not vary with temperature, and were kept fixed at $a_{ij} = 25\ \epsilon_0/r_c$. Eq. 4 produces good solvent conditions for $T = 0.8\ \epsilon_0/k_B$.[2]

In addition to the three DPD forces, adjacent monomers on the polymer chain are connected to each other through forces derived from the finite-extensible nonlinear elastic (FENE) potential, defined as:

$$U_F(r_{i,i+1}) = -\frac{k_F}{2}(r_{max} - r_{eq})^2 \ln\left[1 - \left(\frac{r_{i,i+1} - r_{eq}}{r_{max} - r_{eq}}\right)^2\right] \tag{5}$$

if the distance between adjacent monomers $r_{i,i+1} < r_{max}$, and is infinite otherwise. The bond strength was set to $k_F = 50\ \epsilon_0/r_c^2$, the maximum extension $r_{max} = 2\ r_c$, and the equilibrium bond length $r_{eq} = 0.7\ r_c$. The first monomer of each polymer chain was attached to the substrate by imposing a harmonic potential between a random DPD bead on the surface of the substrate with the monomer at the chain end. The harmonic potential was used to enable simulations with a larger timestep.

Finally, eDPD introduces several heat fluxes to account for energy flow within the system. Each DPD bead i possesses a temperature T_i, the time evolution of which is governed by the equation of motion:

$$C_v \frac{dT_i}{dt} = Q_i = \sum_{i \neq j} (Q_{ij}^C + Q_{ij}^V + Q_{ij}^R) \tag{6}$$

where C_v is the heat capacity of the system and Q_i is the net heat flux of particle i. For each particle i, Q_i is the summation of collisional (Q_{ij}^C), viscous (Q_{ij}^V) and random heat flux (Q_{ij}^R) represented as:

$$Q_{ij}^C = k_{ij} w^D(r_{ij}) \left(\frac{1}{T_i} - \frac{1}{T_j} \right) \tag{7}$$

$$Q_{ij}^V = \frac{1}{2C_v} w^D(r_{ij}) [\gamma_{ij} (\hat{\mathbf{r}}_{ij} \cdot \mathbf{v}_{ij})^2 - \zeta_{ij}^2] - \zeta_{ij} w^R(r_{ij}) (\hat{\mathbf{r}}_{ij} \cdot \mathbf{v}_{ij}) \theta_{ij} \tag{8}$$

and,

$$Q_{ij}^R = \upsilon_{ij} w^R(r_{ij}) (\Delta t)^{-1/2} \Theta_{ij} \tag{9}$$

where Θ_{ij} is a uniform random number of zero mean and unit variance (note: $\Theta_{ij} \neq \theta_{ij}$). The collisional and random heat fluxes are related by their leading coefficients $\upsilon_{ij}^2 = 2k_B k_{ij}$, where k_{ij} is the thermal conductivity represented as $k_{ij} = C_v^2 \kappa (T_i + T_j)^2 / 4k_B$. C_v and κ are the heat capacity and the heat friction coefficient, respectively. We set $C_v = 1000$ and $\kappa = 0.001$. All equations of motion were integrated using the velocity-Verlet algorithm with a time step of $\Delta t = 0.01\tau_0$. τ_0 represents the fundamental unit of time in eDPD.

Initial conditions were prepared by attaching grafted polymers to random positions on the substrate, and randomly orienting the monomers of each chain away from the substrate in the $+z$-direction. The polymers were equilibrated until their mean-squared end-to-end distance stabilized at a constant value, after which 10^6 production time steps at each temperature were performed and monomer positions were collected for analysis by proper orthogonal decomposition. To change the system temperature, we set the temperature of the thermal reservoir T_r to the desired temperature, which resulted in an additional thermal flux into the system for each particle: $Q_i^S(t) = \lambda C_\text{v} [T_r(t) - T_i(t)]$, with $\lambda = 0.1$. This additional heat flux for each particle is added to the right-hand side of Eq. 6.

2 Proper Orthogonal Decomposition (POD)

Proper orthogonal decomposition (POD) was used to observe the relaxation modes of the polymers. During the production stage of the simulations, snapshots of the monomer coordinates were collected after every 500 time steps and post-processed for POD. For each time point, the monomer coordinates were corrected to remove the effects of any net translational motion by subtracting off the instantaneous center of mass location of each polymer chain, i.e.:

$$\tilde{\mathbf{r}}_i(t) = \mathbf{r}_i(t) - \frac{1}{N} \sum_{j=1}^{N} \mathbf{r}_j(t) \tag{10}$$

Three covariance matrices $\mathbf{X}^q$ were formed from $\tilde{\mathbf{r}}_i$ for each of the three spatial dimensions in the system (i.e., $q = x$, y, or z). Each diagonal element ii of the covariance matrix provides a measure of the variance of monomer i's position (i.e., how much it is changing), while the off-diagonal elements measure the covariance (i.e., how statistically related they are). For example, the ij element of $\mathbf{X}$, with $i \neq j$, quantifies how statistically related the i and j monomers of the polymer chain are in time. The covariance matrix is then defined as:

$$\mathbf{X}_{ij}^q = \frac{1}{T} \sum_{t=0}^{T-1} [\tilde{\mathbf{r}}_i(t) \cdot \hat{\mathbf{q}}] [\tilde{\mathbf{r}}_j(t) \cdot \hat{\mathbf{q}}] \tag{11}$$

where T denotes the total number of temporal snapshots that were recorded. Note that we do not subtract off the time-averaged configuration of the monomers in this approach, as is commonly done in many implementations of POD or principal component analysis (PCA). The eigenvectors ψ_p of $\mathbf{X}^q$ are the modes that describe the fundamental displacement patterns of the monomers in the direction of $\hat{\mathbf{q}}$. Each ψ_p is a column vector containing N elements for each of the monomers comprising the polymer

chain. The modes are arranged in order from the lowest mode ($p = 1$) to the highest mode ($p = N$) as a descending series of their eigenvalues λ_p (i.e., mode $p = 1$ has the largest eigenvalue, etc.). As p increases, each mode describes motions of increasingly smaller regions of the polymer chain. More details regarding the POD procedure, and its application to determining relaxation times can be found elsewhere in the literature.[2,7–9] In the present work, we focused exclusively on determining the effect of grafting on the dynamical modes themselves, and not the relaxation times of the modes.

For the case of a free chain, spatial symmetry allows one to average the modes in the x, y, and z-directions. However, grafting polymers to a planar substrate breaks the symmetry of the system in the direction perpendicular to the substrate. For this reason, we separately analyzed the modes in the perpendicular direction (z), and averaged the modes in the parallel directions (x and y).

3 Discrete Eigenmode Analysis of a Perturbed Rouse Operator

To motivate the basis used to represent the POD eigenmodes, we consider a discrete nearest-neighbor model for chain connectivity. The unperturbed operator yields the familiar Rouse modes, while a localized perturbation associated with grafting motivates the inclusion of a surface-localized exponential contribution. For each polymer chain, we describe the configuration of the polymer at time t by the vector

$$\mathbf{R}(t) = \begin{bmatrix}\mathbf{r}_0(t) & \mathbf{r}_1(t) & \cdots & \mathbf{r}_n(t) & \cdots & \mathbf{r}_{N-1}(t)\end{bmatrix}^T \tag{12}$$

3.1 Eigenmodes of the Unperturbed Discrete Rouse Operator

For free linear chains, the equations of motion for the monomers comprising a chain of length N can be cast in terms of a discrete Langevin equation as:

$$\frac{d}{dt}\mathbf{R}(t) = -\frac{3k_BT}{b^2\zeta}\begin{bmatrix} 1 & -1 & 0 & 0 & \cdots & 0 \\ -1 & 2 & -1 & 0 & \cdots & 0 \\ 0 & -1 & 2 & -1 & \cdots & 0 \\ \vdots & \ddots & \ddots & \ddots & \ddots & \vdots \\ 0 & 0 & 0 & \ldots & -1 & 1 \end{bmatrix}\mathbf{R}(t) + \begin{bmatrix}\mathbf{f}_0 \\ \mathbf{f}_1 \\ \mathbf{f}_2 \\ \vdots \\ \mathbf{f}_{N-1}\end{bmatrix} \tag{13}$$

or equivalently,

$$\frac{d}{dt}\mathbf{R}(t) = -\frac{3k_BT}{b^2\zeta}\mathbf{K}\mathbf{R}(t) + \mathbf{F}(t) \tag{14}$$

where the discrete Rouse operator $\mathbf{K}$ (or connectivity matrix) represents the discretized form of Hooke's law and the connectivity of a linear chain (i.e., that monomer 1 is connected to monomer 2, that monomer 2 is connected to monomers 1 and 3, and so on). b and ζ are the statistical segment length and friction coefficient for the polymer, respectively. Note that $\mathbf{K}$ is identical in form to the discrete Laplacian operator. The thermal noise $\mathbf{F}(t)$ determines the stochastic amplitudes of the modes, but does not alter the eigenvectors of $\mathbf{K}$. The classical Rouse modes ϕ_p can be obtained by solving the eigenvalue problem $\mathbf{K}\phi = \lambda\phi$. Examining the elements corresponding to an interior monomer n,

$$(\mathbf{K}\phi)_n = 2\phi_n - \phi_{n-1} - \phi_{n+1} = \lambda\phi_n \tag{15}$$

giving rise to a recurrence relation:

$$\phi_{n+1} + \phi_{n-1} = (2 - \lambda)\phi_n \tag{16}$$

As in the solution of linear differential equations with constant coefficients, exponential functions are natural trial solutions because they are eigenfunctions of differentiation. Likewise, the geometric sequence r^n is an eigenfunction of the discrete shift operator, since shifting the sequence simply multiplies it by r. For this reason, the analogous trial solution is $\phi_n = r^n$. With this trial solution, we obtain a characteristic polynomial

$$r^2 - (2 - \lambda)r + 1 = 0 \tag{17}$$

with solutions:

$$r = \frac{(2 - \lambda) \pm \sqrt{(2 - \lambda)^2 - 4}}{2} \tag{18}$$

The functional form of ϕ will depend on the value of λ (i.e., whether r is real or complex in Eq. 18). First,

$$\phi^T \mathbf{K} \phi = \sum_{n=0}^{N-2} (\phi_{n+1} - \phi_n)^2 \geq 0 \tag{19}$$

implying that $\lambda \geq 0$. Second,

$$(\phi_{n+1} - \phi_n)^2 \leq 2\phi_{n+1}^2 + 2\phi_n^2 \tag{20}$$

implying that $\phi^T \mathbf{K} \phi \leq 4\phi^T \phi$. Therefore, $0 \leq \lambda \leq 4$ for the unperturbed discrete Rouse chain and the roots are complex conjugates. This condition on λ produces an oscillatory solution $\phi_n = Ae^{ikn} + Be^{-ikn}$, with A and B determined by the boundary conditions:

$$\left(\frac{\partial \phi_p}{\partial s}\right)_{s=0} = \left(\frac{\partial \phi_p}{\partial s}\right)_{s=1} = 0. \tag{21}$$

By allowing only discrete values of $k = k_p = p\pi/N$, the eigenvalues are:

$$\lambda_p = 4\sin^2\left(\frac{\pi p}{2N}\right) \tag{22}$$

and

$$\phi_p = \sqrt{\frac{2}{N}} \cos\left[\frac{\pi p}{N}\left(n + \frac{1}{2}\right)\right] \approx \sqrt{\frac{2}{N}} \cos(\pi p s) \tag{23}$$

where $s = n/N$. This is the classical result from Rouse. Although our DPD simulations use a FENE potential in place of the harmonic potential used above, we have shown previously[2,8,9] that POD identifies dynamical modes for free linear chains that are in quantitative agreement with those derived here.

3.2 Surface-Localized Solution of a Perturbed Operator

Grafting a polymer to a substrate breaks the symmetry of the Rouse model. As a minimal, perturbative model to explain the observed dynamical modes in the z-direction (perpendicular to the substrate), we assume that grafting the end of a polymer chain perturbs the discrete operator to create an effective operator $\mathbf{K} \to \mathbf{K} + \delta\mathbf{K}$. We further assume that the effect of the grafting condition is not felt sufficiently far from the grafted end along the chain contour, so that for large values of s, $\delta\mathbf{K} \to 0$. This condition also applies in the x- and y-directions for all values of s, resulting in a recovery of the standard Rouse modes in the parallel directions. Note that we do not specify the exact form of $\delta\mathbf{K}$.

Near the substrate, the eigenvalue problem becomes $(\mathbf{K} + \delta\mathbf{K})\psi = \lambda_{loc}\psi$ where $\psi \neq \phi$. Far from the substrate, since $\delta\mathbf{K} \to 0$, we obtain a recurrence relation that is similar to that for the unperturbed Rouse operator, i.e.,

$$[(\mathbf{K} + \delta\mathbf{K})\psi]_n \approx 2\psi_n - \psi_{n+1} - \psi_{n-1} = \lambda_{loc}\psi_n \tag{24}$$

and a characteristic polynomial

$$r^2 - (2 - \lambda_{loc})r + 1 = 0 \tag{25}$$

with solutions of the form in Eq. 18. We are interested in a surface-localized solution whose magnitude vanishes with increasing contour distance from the grafted end. Such a solution requires a characteristic root satisfying $|r| < 1$. For the bulk recurrence, this occurs when the corresponding eigenvalue lies outside the extended-mode band, $\lambda_{loc} < 0$ or $\lambda_{loc} > 4$. For $\lambda_{loc} > 4$, this gives a staggered profile $(-1)^n e^{\pm kn}$ and is not consistent with the modes observed by POD. For $\lambda_{loc} < 0$, we obtain $\psi_n = Ae^{\kappa n} + Be^{-\kappa n}$, with $A = 0$ because of the requirement that the effects of the perturbation decay as n (or s) increases. Finally, we re-express the localized contribution $\psi_n^{loc} \propto e^{-n/\xi_n}$, where ξ_n is a localization length. The eigenvalue $\lambda_{loc} = 2 - 2\cosh(\xi_n^{-1})$.

Because the instantaneous center-of-mass coordinate is removed from every chain configuration before constructing the POD covariance matrix, each POD mode with a nonzero eigenvalue must have zero contour average, i.e.,

$$\psi_n^{loc} = e^{-n/\xi_n} - \frac{1}{N}\sum_{m=0}^{N-1} e^{-m/\xi_n} \tag{26}$$

resulting in a final surface-localized contribution of

$$\psi_n^{loc} = e^{-s/\xi} - \left\langle e^{-s/\xi} \right\rangle_s \tag{27}$$

where $\langle \cdots \rangle_s$ denotes the average over s.

3.3 Reduced Basis Representation of the POD Modes

The derivations in the preceding section motivate the functional form of the POD modes we observed, rather than providing an exact derivation of them. The nontranslational Rouse modes ϕ_p form a complete orthonormal basis for chain deformations with zero contour average. Because the instantaneous center of mass motion has been removed, every POD mode has zero contour average and may be expanded as a linear combination of ϕ_p $(p > 0)$ so that:

$$\psi_m = A_{exp}\left[e^{-s/\xi} - \left\langle e^{-s/\xi}\right\rangle_s\right] + \sum_{p=1}^{p_{max}} A_p \phi_p \tag{28}$$

where ξ is the fraction of the chain contour over which the perturbation is felt, and p_{max} denotes the number of Rouse modes retained in the reduced representation. Because the Rouse modes satisfy the Neumann boundary conditions in Eq. 21,

$$\left(\frac{\partial \psi_m}{\partial s}\right)_{s=0} = -\frac{A_{exp}}{\xi}. \tag{29}$$

Thus, the nonzero boundary slope is supplied entirely by the localized exponential contribution. The parameter ξ sets the characteristic contour distance over which this graft-induced contribution decays and the mode approaches bulk Rouse-like behavior. As discussed in the main text, we verified that the slope of the POD mode near $s = 0$ is consistent with $-A_{\mathrm{exp}}/\xi$.

The coupling of multiple Rouse modes in the first perpendicular POD mode can be rationalized by again considering the eigenvalue problem solved in previous sections. Grafting the polymer to the substrate perturbs the Rouse operator such that $\mathbf{K} \to \mathbf{K} + \delta\mathbf{K}$, with $\delta\mathbf{K} \to 0$ far from the substrate. The eigenvalue problem becomes:

$$(\mathbf{K} + \delta\mathbf{K})\psi_m = \Lambda_m \psi_m \tag{30}$$

Expanding ψ_m in the Rouse basis:

$$\psi_m = \sum_{p=1}^{N} A_p \phi_p \tag{31}$$

Substituting this expansion into the eigenvalue problem yields

$$\mathbf{K}\sum_{p=1}^{N} A_p\phi_p + \delta\mathbf{K}\sum_{p=1}^{N} A_p\phi_p = \Lambda_m \sum_{p=1}^{N} A_p\phi_p \tag{32}$$

Mode coupling in the case of $\delta\mathbf{K} \neq 0$ can be observed by projecting the perturbed eigenvalue equation onto the Rouse mode ϕ_q:

$$\begin{aligned}
\phi_q^T(\mathbf{K} + \delta\mathbf{K})\sum_{p=1}^{N} A_p\phi_p &= \Lambda_m \sum_{p=1}^{N} A_p \phi_q^T \phi_p \\
&= \sum_{p=1}^{N} A_p \phi_q^T \mathbf{K}\phi_p + \sum_{p=1}^{N} A_p \phi_q^T \delta\mathbf{K}\phi_p \\
&= \lambda_q A_q + \sum_{p=1}^{N} A_p \phi_q^T \delta\mathbf{K}\phi_p \\
&= \Lambda_m A_q.
\end{aligned} \tag{33}$$

since $\phi_q^T \mathbf{K}\phi_p = \lambda_q \delta_{qp}$ due to the orthonormal property of the Rouse modes (δ_{qp} is the Kronecker delta). Therefore,

$$(\Lambda_m - \lambda_q)A_q = \sum_{p=1}^{N} A_p \phi_q^T \delta\mathbf{K}\phi_p = \sum_{p=1}^{N} \delta K_{qp} A_p \tag{34}$$

where $\delta K_{qp} = \phi_q^T \delta\mathbf{K}\phi_p$ are the mode coupling matrix elements. The diagonal elements δK_{pp} shift (renormalize) the Rouse eigenvalues, whereas the off-diagonal elements couple Rouse modes of different indices. If $\delta\mathbf{K} = 0$, then $\delta K_{qp} = 0$, $\Lambda_m = \lambda_q$ and only a single Rouse mode appears in ψ_m (i.e., ϕ_q). This

is the behavior observed in the unperturbed Rouse model. However, if $\delta\mathbf{K} \neq 0$, then the off-diagonal elements δK_{qp} are generally non-zero and the eigenmodes of the perturbed operator become hybridized linear combinations of the Rouse modes. As we will show in the next section, we observed that while mode coupling does occur in ψ, the perpendicular POD mode is dominated by the $p = 1$ Rouse mode, with increasingly smaller contributions from higher-order Rouse modes ($p > 1$).

Although a localized contribution (e.g., from grafting) can formally be represented by the complete Rouse basis as in Eq. 33, doing so requires many higher-order Rouse modes. Introducing the projected exponential in Eq. 28 as an additional reduced-basis function provides a compact reduced representation of the localized boundary structure. In addition, the dominant collective fluctuations are expected to be represented primarily by the lowest-order Rouse modes,[10] while higher-order modes describe progressively shorter wavelength fluctuations that contribute only weakly to the observed POD mode. Indeed, we observed that A_p decreases as p increases, and that contributions beyond $p = 5$ produced negligible effects on our fitting and, therefore, we kept $p_{max} = 5$. The projected exponential is not generally orthogonal to the truncated Rouse basis, so the amplitudes of the terms in Eq. 28 are determined simultaneously through least-squares optimization. The fitting parameters are $\{A_{exp}, A_1, A_2, \ldots, A_{p_{max}}, \xi\}$.

We quantified the relative contribution of the localized mode to the Rouse mode contributions by the ratio:

$$f_{loc} = A_{exp} \Bigg/ \sum_{p=1}^{p_{max}} |A_p| \tag{35}$$

4 Polymer Brush Simulation Results

4.1 Fitting Results

4.1.1 Parallel Direction

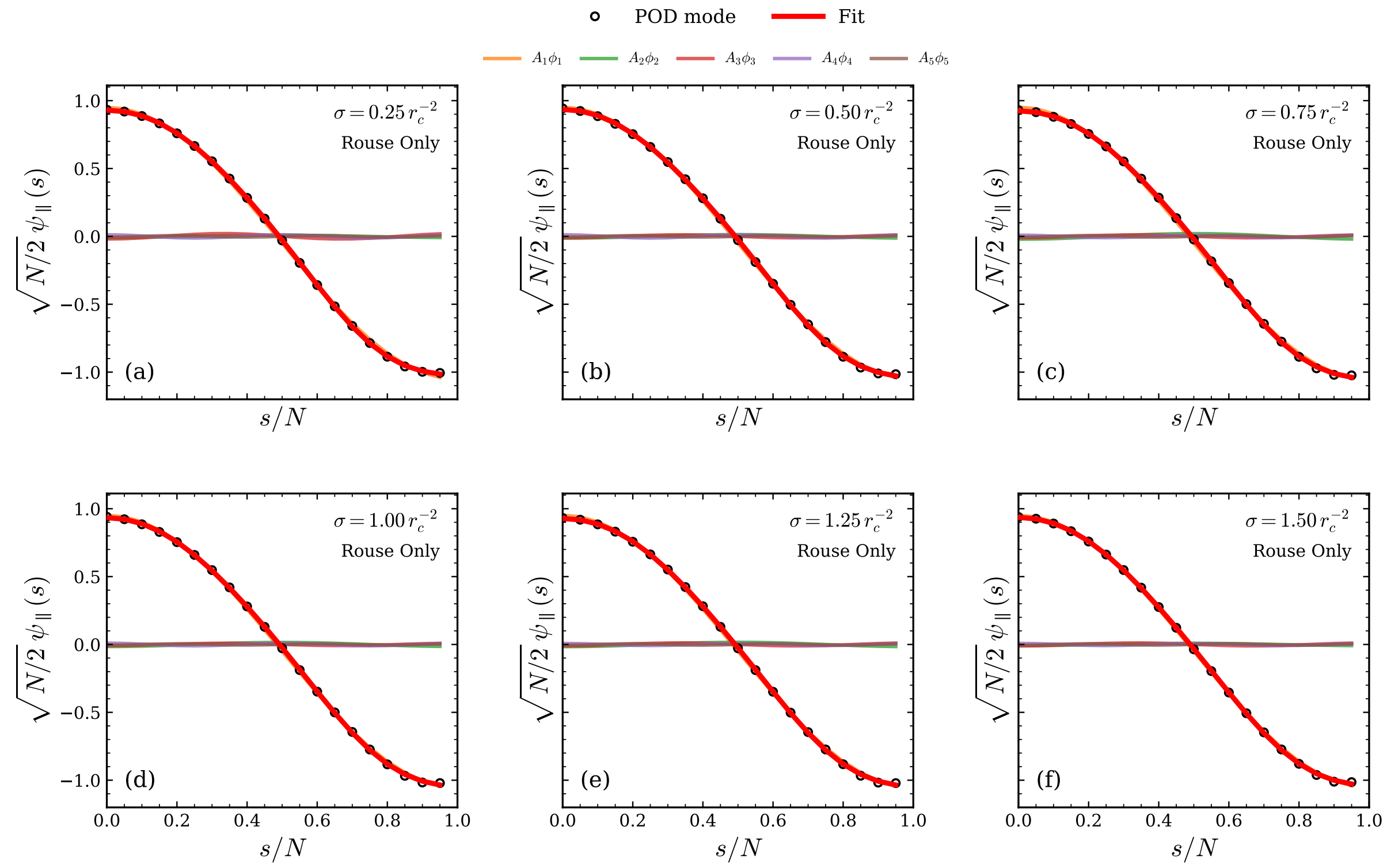

Figure S1: Fits of the parallel modes $\psi_{\parallel}(s)$ for planar brushes at $T = 0.8\ \epsilon_0/k_B$ and $N = 20$ for grafting densities of (a) $\sigma = 0.25\ r_c^{-2}$, (b) $\sigma = 0.50\ r_c^{-2}$, (c) $\sigma = 0.75\ r_c^{-2}$, (d) $\sigma = 1.00\ r_c^{-2}$, (e) $\sigma = 1.25\ r_c^{-2}$, and (f) $\sigma = 1.50\ r_c^{-2}$. The $p = 1$ POD mode is shown as points, and the fit as a solid red line. The dashed line shows the localized mode, and colored lines the contribution of higher-order Rouse modes. f_{loc} denotes the relative contribution of the localized mode to the Rouse modes.

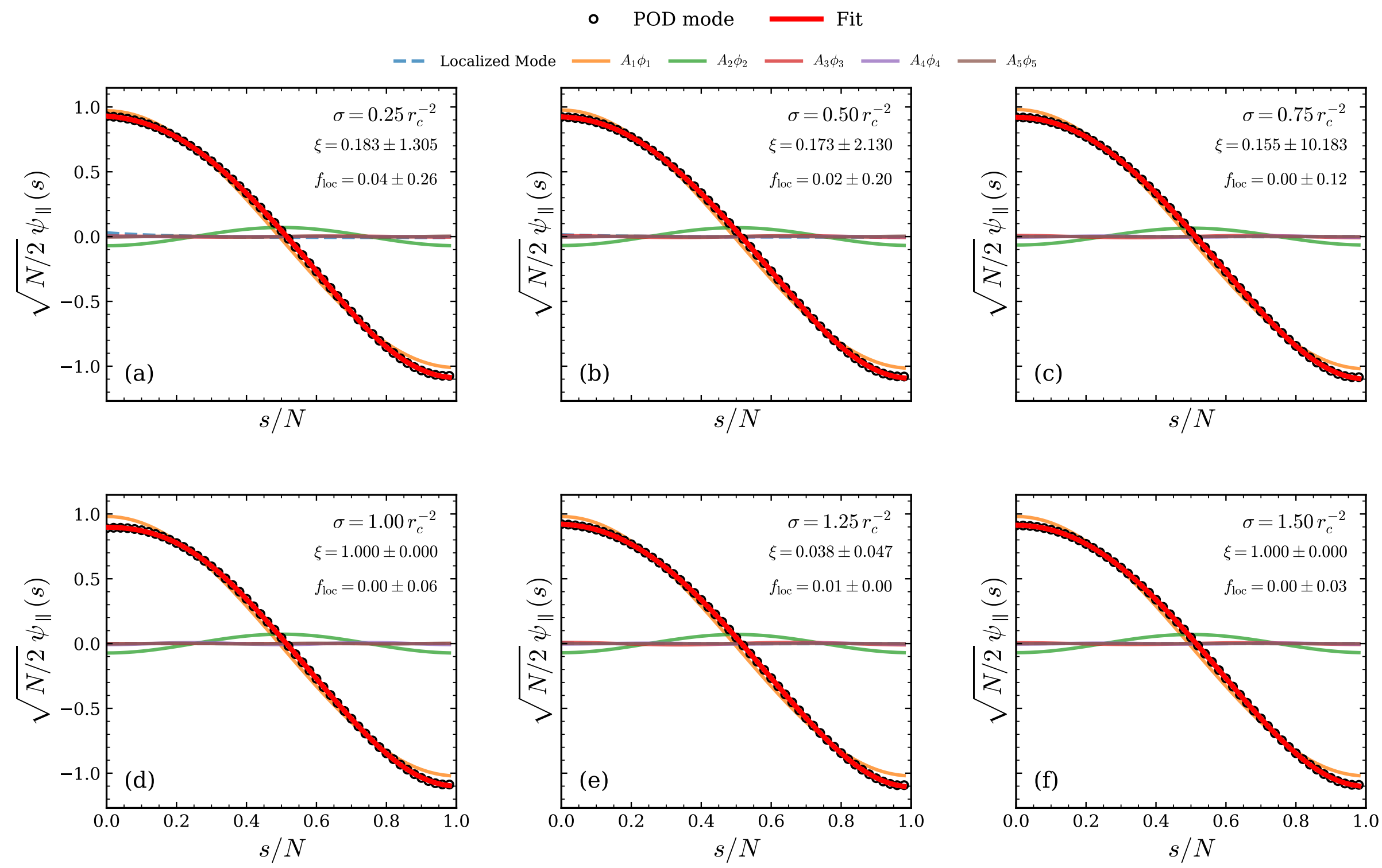

Figure S2: Fits of the parallel modes $\psi_\parallel(s)$ for planar brushes at $T = 0.8\ \epsilon_0/k_B$ and $N = 50$ for grafting densities of (a) $\sigma = 0.25\ r_c^{-2}$, (b) $\sigma = 0.50\ r_c^{-2}$, (c) $\sigma = 0.75\ r_c^{-2}$, (d) $\sigma = 1.00\ r_c^{-2}$, (e) $\sigma = 1.25\ r_c^{-2}$, and (f) $\sigma = 1.50\ r_c^{-2}$. The $p = 1$ POD mode is shown as points, and the fit as a solid red line. The dashed line shows the localized mode, and colored lines the contribution of higher-order Rouse modes. f_{loc} denotes the relative contribution of the localized mode to the Rouse modes.

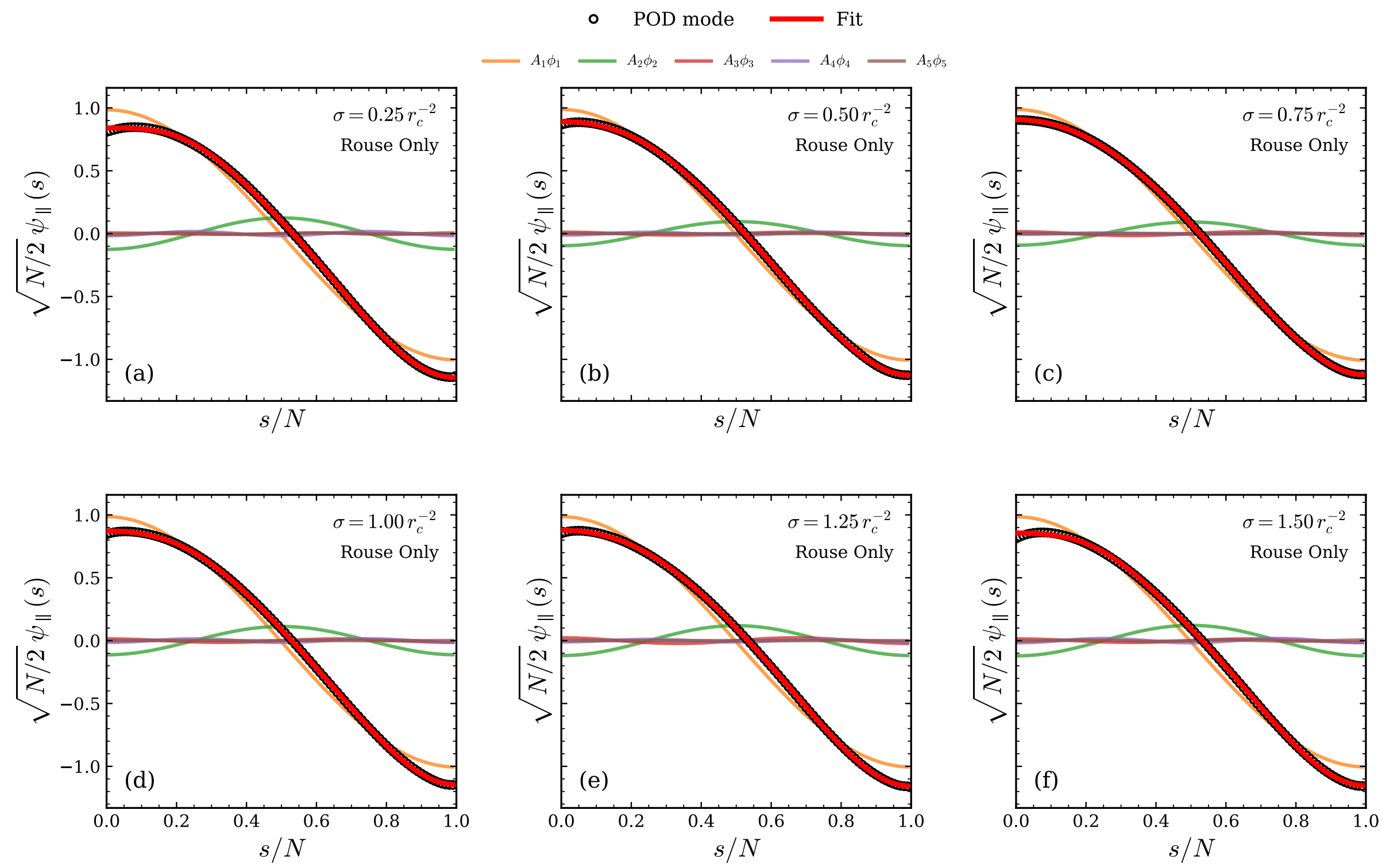

Figure S3: Fits of the parallel modes $\psi_\parallel(s)$ for planar brushes at $T = 0.8\ \epsilon_0/k_B$ and $N = 100$ for grafting densities of (a) $\sigma = 0.25\ r_c^{-2}$, (b) $\sigma = 0.50\ r_c^{-2}$, (c) $\sigma = 0.75\ r_c^{-2}$, (d) $\sigma = 1.00\ r_c^{-2}$, (e) $\sigma = 1.25\ r_c^{-2}$, and (f) $\sigma = 1.50\ r_c^{-2}$. The $p = 1$ POD mode is shown as points, and the fit as a solid red line. The dashed line shows the localized mode, and colored lines the contribution of higher-order Rouse modes. f_{loc} denotes the relative contribution of the localized mode to the Rouse modes.

4.1.2 Perpendicular Direction

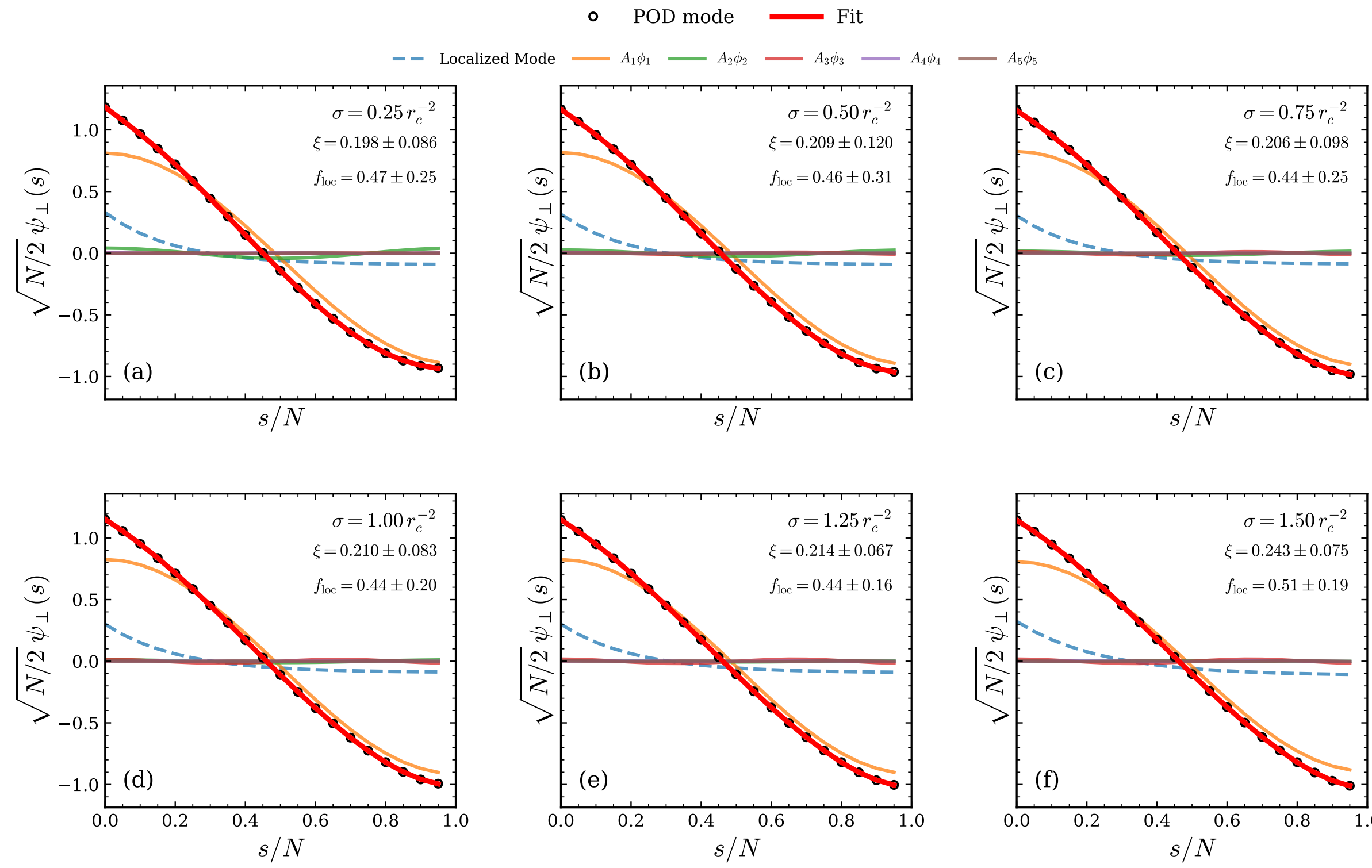

Figure S4: Fits of the perpendicular modes $\psi_\perp(s)$ for planar brushes at $T = 0.8\ \epsilon_0/k_B$ and $N = 20$ for grafting densities of (a) $\sigma = 0.25\ r_c^{-2}$, (b) $\sigma = 0.50\ r_c^{-2}$, (c) $\sigma = 0.75\ r_c^{-2}$, (d) $\sigma = 1.00\ r_c^{-2}$, (e) $\sigma = 1.25\ r_c^{-2}$, and (f) $\sigma = 1.50\ r_c^{-2}$. The $p = 1$ POD mode is shown as points, and the fit as a solid red line. The dashed line shows the localized mode, and colored lines the contribution of higher-order Rouse modes. f_{loc} denotes the relative contribution of the localized mode to the Rouse modes.

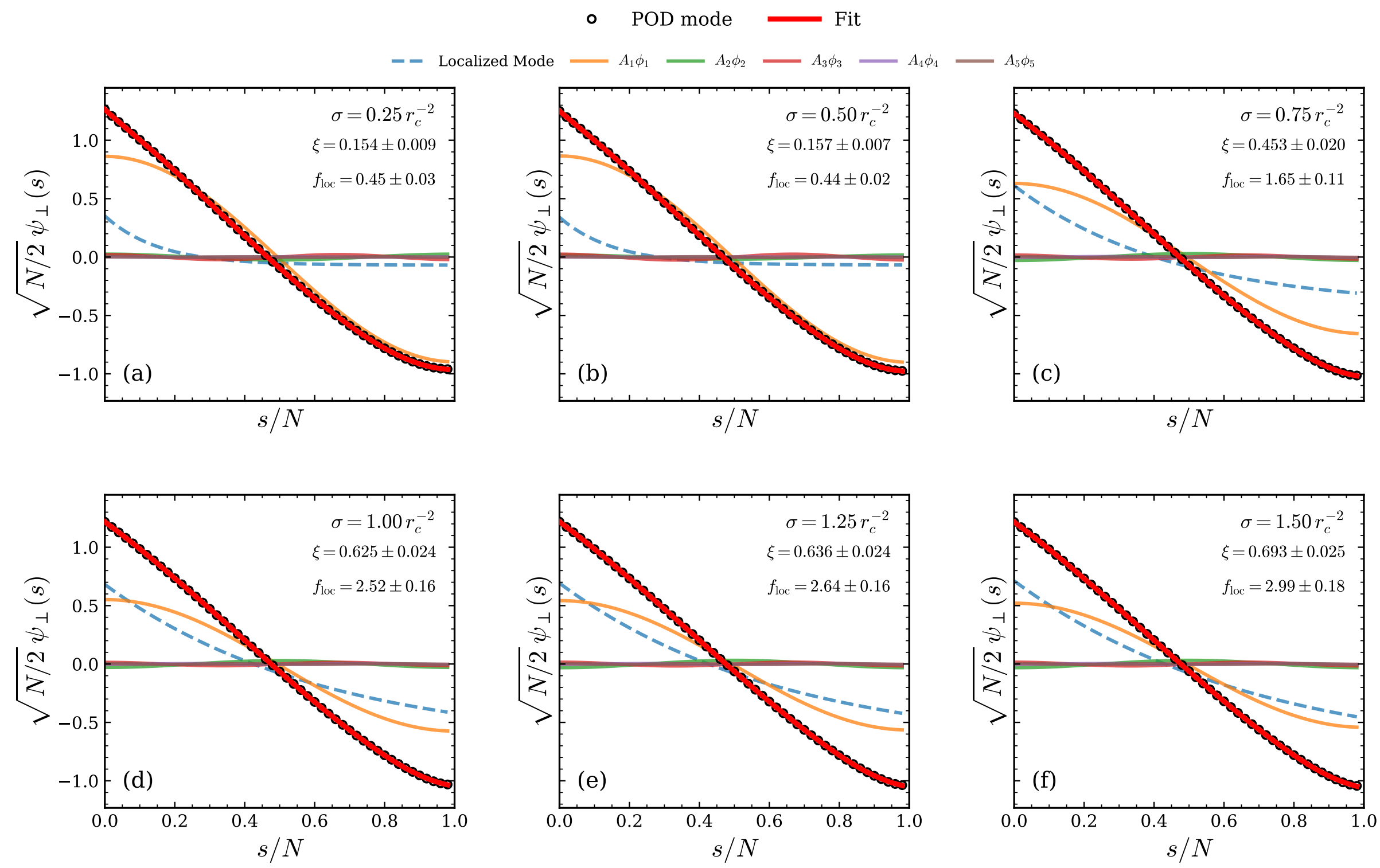

Figure S5: Fits of the perpendicular modes $\psi_\perp(s)$ for planar brushes at $T = 0.8\ \epsilon_0/k_B$ and $N = 50$ for grafting densities of (a) $\sigma = 0.25\ r_c^{-2}$, (b) $\sigma = 0.50\ r_c^{-2}$, (c) $\sigma = 0.75\ r_c^{-2}$, (d) $\sigma = 1.00\ r_c^{-2}$, (e) $\sigma = 1.25\ r_c^{-2}$, and (f) $\sigma = 1.50\ r_c^{-2}$. The $p = 1$ POD mode is shown as points, and the fit as a solid red line. The dashed line shows the localized mode, and colored lines the contribution of higher-order Rouse modes. f_{loc} denotes the relative contribution of the localized mode to the Rouse modes.

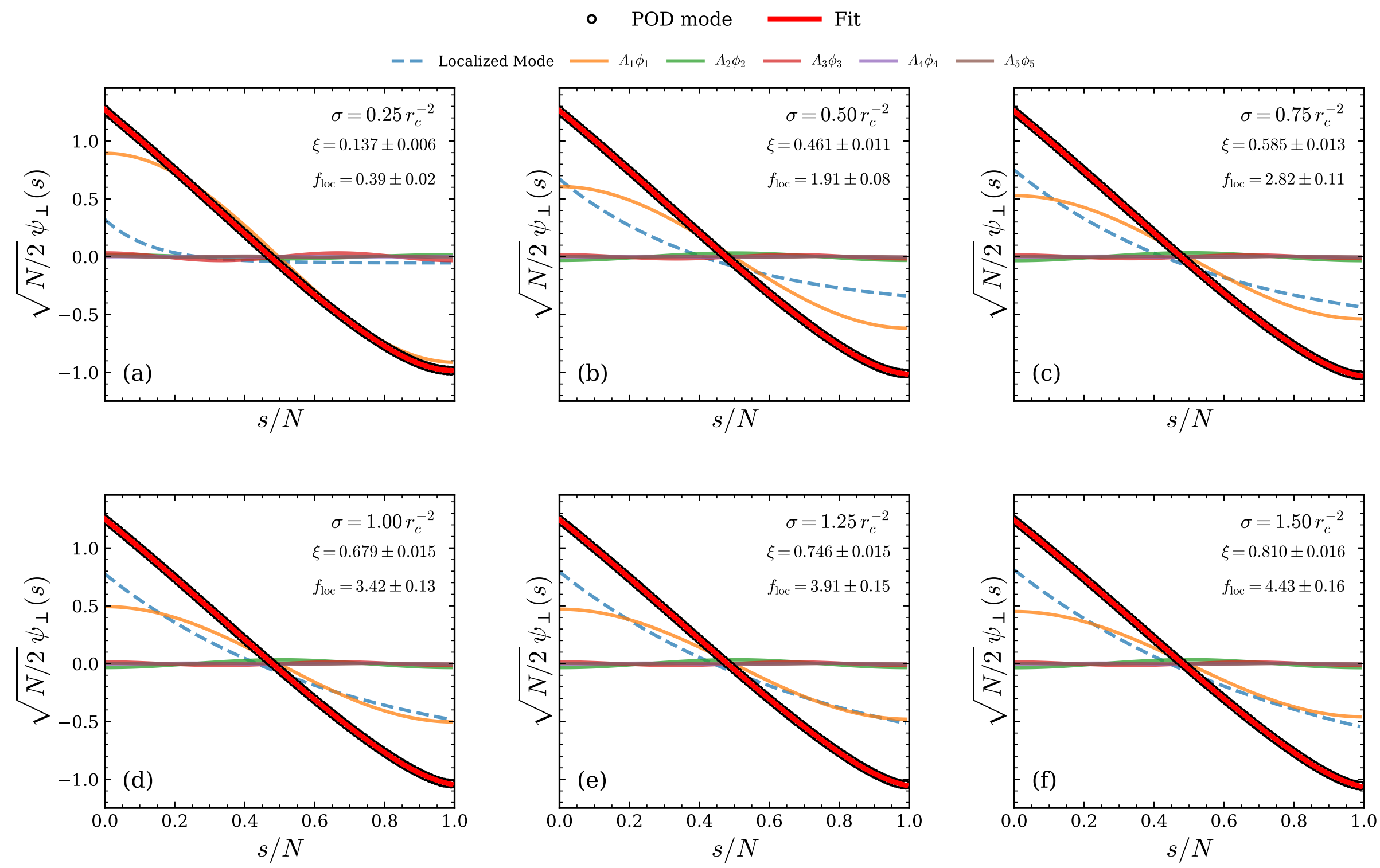

Figure S6: Fits of the perpendicular modes $\psi_\perp(s)$ for planar brushes at $T = 0.8\ \epsilon_0/k_B$ and $N = 100$ for grafting densities of (a) $\sigma = 0.25\ r_c^{-2}$, (b) $\sigma = 0.50\ r_c^{-2}$, (c) $\sigma = 0.75\ r_c^{-2}$, (d) $\sigma = 1.00\ r_c^{-2}$, (e) $\sigma = 1.25\ r_c^{-2}$, and (f) $\sigma = 1.50\ r_c^{-2}$. The $p = 1$ POD mode is shown as points, and the fit as a solid red line. The dashed line shows the localized mode, and colored lines the contribution of higher-order Rouse modes. f_{loc} denotes the relative contribution of the localized mode to the Rouse modes.

4.2 Summary of Reduced Basis Fitting Parameters

4.2.1 $N = 20$

Table S1: Parameters obtained from least-squares fitting of the parallel POD modes for $N = 20$. For all fits reported here, the surface-localized contribution was omitted, i.e., $A_{\mathrm{exp}} = 0$, and only the Rouse basis functions were included.

$\sigma\,(r_c^{-2})$	A_1	A_2	A_3	A_4	A_5	R^2	RMSE
0.25	1.001620	-0.009091	-0.020349	0.012384	-0.005938	0.999976	0.003478
0.50	1.002153	-0.013219	-0.011371	0.012622	-0.005356	0.999941	0.005428
0.75	1.003001	-0.020969	-0.011151	0.009515	-0.006177	0.999939	0.005521
1.00	1.002659	-0.015410	-0.008831	0.010070	-0.004693	0.999936	0.005657
1.25	1.002894	-0.016407	-0.011217	0.007922	-0.005518	0.999947	0.005144
1.50	1.002261	-0.008805	-0.010974	0.008223	-0.005197	0.999950	0.005016

Table S2: Parameters obtained from least-squares fitting of the perpendicular POD modes for $N = 20$.

$\sigma\,(r_c^{-2})$	ξ	A_{exp}	A_1	A_2	A_3	A_4	A_5	f_{loc}	R^2	RMSE
0.25	0.197785	0.423893	0.854137	0.041289	0.000747	0.002523	-0.001625	0.470824	0.999998	0.000896
0.50	0.209082	0.410244	0.859427	0.026139	0.008394	0.001546	-0.000483	0.457868	0.999998	0.000981
0.75	0.205605	0.393643	0.867617	0.016749	0.012972	0.000098	0.000861	0.438211	0.999999	0.000815
1.00	0.209659	0.390815	0.868805	0.011204	0.015817	-0.000832	0.001786	0.434990	0.999999	0.000644
1.25	0.214367	0.393072	0.868119	0.005906	0.017783	-0.001753	0.002480	0.438676	1.000000	0.000486
1.50	0.242891	0.438117	0.849191	-0.001305	0.017854	-0.002677	0.002782	0.501388	1.000000	0.000469

4.2.2 $N = 50$

Table S3: Parameters obtained from least-squares fitting of the parallel POD modes for $N = 50$. For all fits reported here, the surface-localized contribution was omitted, i.e., $A_{\mathrm{exp}} = 0$, and only the Rouse basis functions were included.

$\sigma\,(r_c^{-2})$	A_1	A_2	A_3	A_4	A_5	R^2	RMSE
0.25	1.000769	-0.064652	0.007206	0.002319	-0.001776	0.999982	0.002977
0.50	1.000842	-0.066024	0.008539	-0.000361	-0.002193	0.999986	0.002626
0.75	1.000992	-0.064965	0.008641	-0.003771	-0.000296	0.999991	0.002087
1.00	1.000861	-0.044203	0.016734	0.001784	0.004829	0.999981	0.003060
1.25	1.000958	-0.060321	0.012783	-0.000880	0.002058	0.999992	0.001952
1.50	1.000849	-0.070191	0.006624	-0.003415	-0.000557	0.999996	0.001489

Table S4: Parameters obtained from least-squares fitting of the perpendicular POD modes for $N = 50$.

$\sigma\,(r_c^{-2})$	ξ	A_{exp}	A_1	A_2	A_3	A_4	A_5	f_{loc}	R^2	RMSE
0.25	0.154355	0.420932	0.880028	0.024870	0.021674	0.001234	0.001549	0.452929	1.000000	0.000428
0.50	0.157044	0.407875	0.883562	0.017952	0.024976	0.000267	0.002552	0.438902	1.000000	0.000313
0.75	0.453052	1.040810	0.643381	-0.028974	0.017440	-0.004151	0.002686	1.494060	0.999999	0.000818
1.00	0.625449	1.381535	0.563184	-0.029039	0.014507	-0.003530	0.002479	2.254688	0.999998	0.000922
1.25	0.636056	1.418174	0.554257	-0.031590	0.016206	-0.004059	0.002662	2.329558	0.999998	0.000934
1.50	0.692858	1.537794	0.531771	-0.031536	0.016023	-0.004038	0.002630	2.624228	0.999998	0.000936

4.2.3 $N = 100$

Table S5: Parameters obtained from least-squares fitting of the parallel POD modes for $N = 100$. For all fits reported here, the surface-localized contribution was omitted, i.e., $A_{\text{exp}} = 0$, and only the Rouse basis functions were included.

$\sigma\,(r_c^{-2})$	A_1	A_2	A_3	A_4	A_5	R^2	RMSE
0.25	0.994691	-0.125356	0.005066	-0.017183	-0.006364	0.999924	0.006171
0.50	0.997446	-0.096054	0.011807	-0.011794	-0.000285	0.999950	0.005018
0.75	0.997681	-0.092262	0.015837	-0.005847	0.001390	0.999995	0.001591
1.00	0.995857	-0.112514	0.013784	-0.014470	-0.001087	0.999953	0.004840
1.25	0.995276	-0.119081	0.022520	-0.010484	-0.000083	0.999959	0.004512
1.50	0.995082	-0.121391	0.013814	-0.017285	-0.004599	0.999871	0.008018

Table S6: Parameters obtained from least-squares fitting of the perpendicular POD modes for $N = 100$.

$\sigma\,(r_c^{-2})$	ξ	A_{exp}	A_1	A_2	A_3	A_4	A_5	f_{loc}	R^2	RMSE
0.25	0.136820	0.374336	0.903612	0.018242	0.033223	0.000230	0.004804	0.389889	0.999999	0.000554
0.50	0.461187	1.141645	0.612291	-0.031941	0.018429	-0.004126	0.002738	1.705156	0.999999	0.000815
0.75	0.585080	1.450580	0.533461	-0.034073	0.016355	-0.004302	0.002614	2.455258	0.999998	0.000952
1.00	0.679124	1.639920	0.498663	-0.033551	0.015728	-0.004092	0.002533	2.957119	0.999998	0.000979
1.25	0.746366	1.781233	0.475848	-0.033901	0.015645	-0.004008	0.002490	3.348860	0.999998	0.000966
1.50	0.810232	1.921037	0.455142	-0.034684	0.015122	-0.004108	0.002421	3.755868	0.999998	0.000964

5 Single Chain Control Simulation Results

To establish the limiting behavior in the absence of interchain crowding, additional simulations were performed for isolated grafted chains. For consistency with the brush systems, these simulations are assigned an effective grafting density of $\sigma = 0.0017\ r_c^{-2}$, although they should be regarded as single-chain controls rather than members of the brush series. The POD modes were analyzed using the same procedure described above. A characteristic surface-normal mode remains present for the isolated grafted chains, demonstrating that its emergence does not require interchain crowding. The fitted parameters exhibit greater statistical uncertainty and do not necessarily extrapolate smoothly from the brush results, indicating that crowding modifies the quantitative characteristics of the mode once a brush is formed.

5.1 Fitting Results

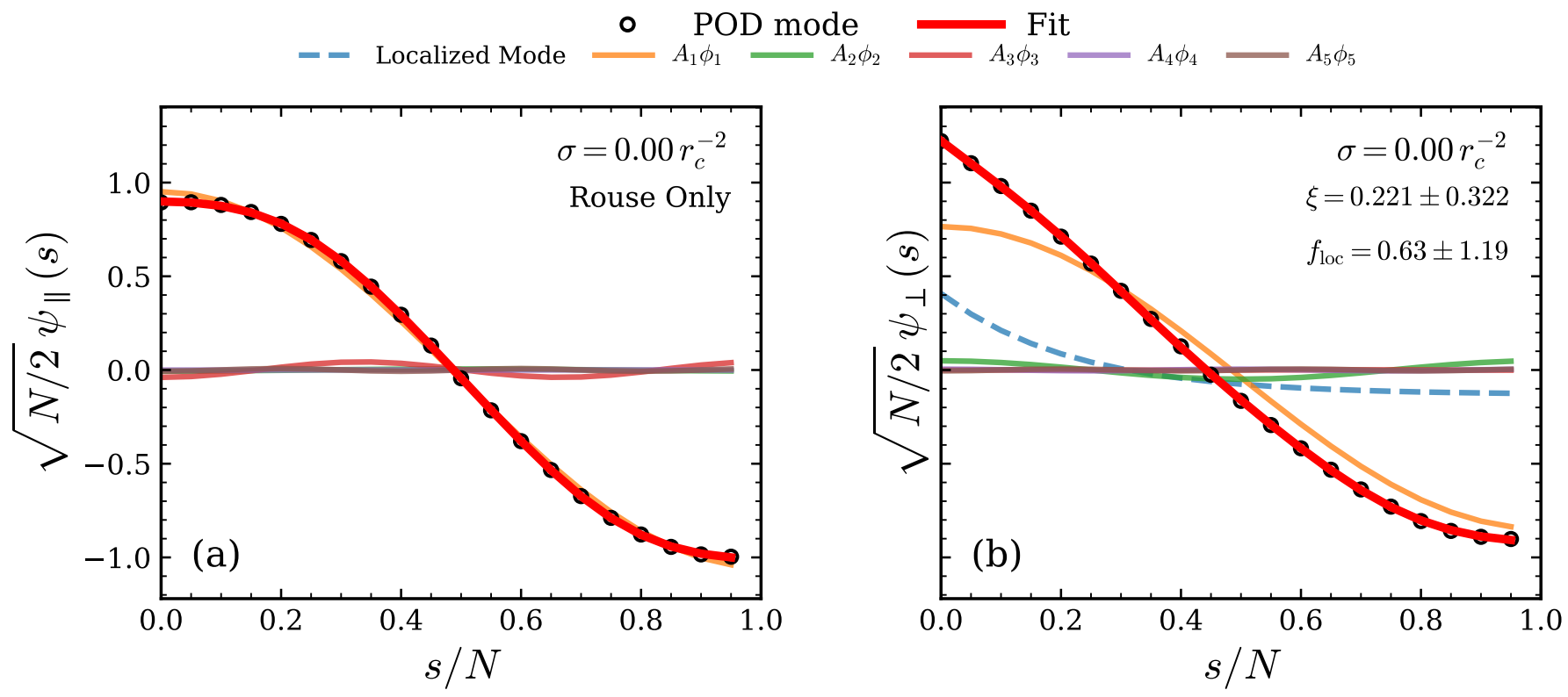

Figure S7: Fits of the (a) parallel and (b) perpendicular modes $\psi_\perp(s)$ for a single grafted chain of length $N = 20$ at $T = 0.8\ \epsilon_0/k_B$. The $p = 1$ POD mode is shown as points, and the fit as a solid red line. The dashed line shows the localized mode, and colored lines the contribution of higher-order Rouse modes. f_{loc} denotes the relative contribution of the localized mode to the Rouse modes.

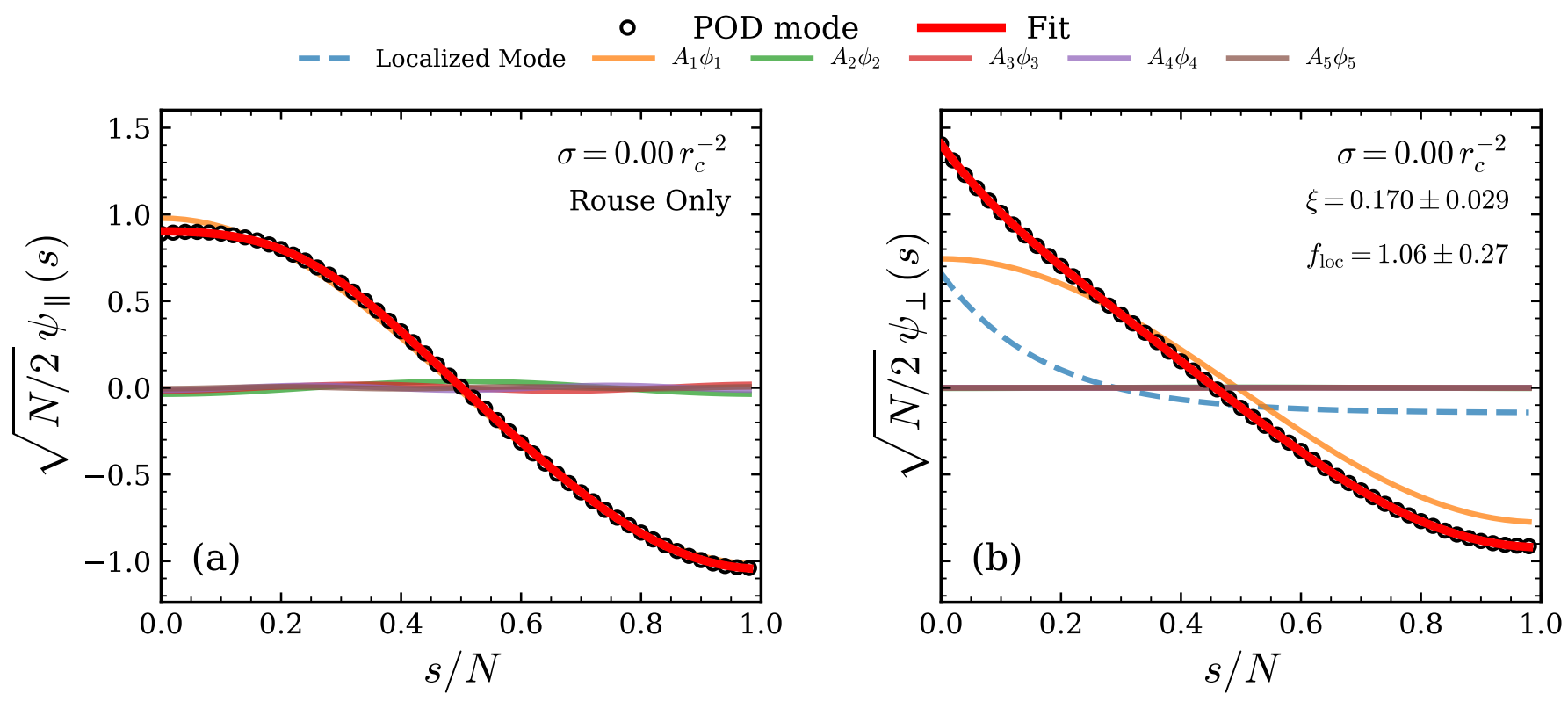

Figure S8: Fits of the (a) parallel and (b) perpendicular modes $\psi_\perp(s)$ for a single grafted chain of length $N = 50$ at $T = 0.8\ \epsilon_0/k_B$. The $p = 1$ POD mode is shown as points, and the fit as a solid red line. The dashed line shows the localized mode, and colored lines the contribution of higher-order Rouse modes. f_{loc} denotes the relative contribution of the localized mode to the Rouse modes.

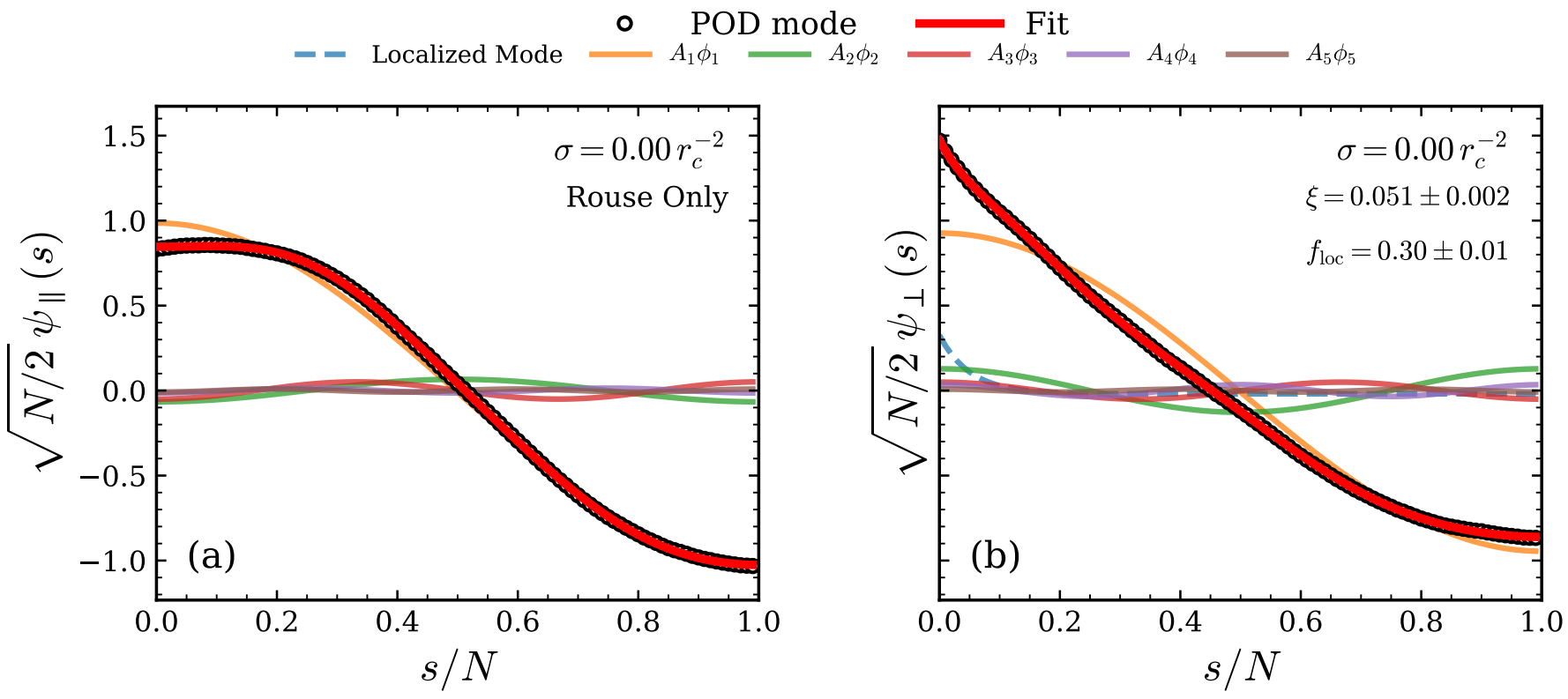

Figure S9: Fits of the (a) parallel and (b) perpendicular modes $\psi_\perp(s)$ for a single grafted chain of length $N = 100$ at $T = 0.8\ \epsilon_0/k_B$. The $p = 1$ POD mode is shown as points, and the fit as a solid red line. The dashed line shows the localized mode, and colored lines the contribution of higher-order Rouse modes. f_{loc} denotes the relative contribution of the localized mode to the Rouse modes.

5.2 Summary of Reduced Basis Fitting Parameters

Table S7: Parameters obtained from least-squares fitting of the parallel POD modes for single chain simulations. For all fits reported here, the surface-localized contribution was omitted, i.e., $A_{\rm exp} = 0$, and only the Rouse basis functions were included.

N	A_1	A_2	A_3	A_4	A_5	R^2	RMSE
20	1.002002	-0.013405	-0.047125	-0.002618	-0.008095	0.999969	0.003966
50	0.999704	-0.056516	-0.027128	-0.0146229	-0.008119	0.999910	0.006685
100	0.998752	-0.026331	-0.041300	-0.001507	-0.002884	0.999784	0.010379

Table S8: Parameters obtained from least-squares fitting of the perpendicular POD modes for single chain simulations.

N	ξ	$A_{\rm exp}$	A_1	A_2	A_3	A_4	A_5	$f_{\rm loc}$	R^2	RMSE
20	0.202634	0.402050	0.849380	0.085057	0.008182	0.008268	-0.007650	0.426246	0.999995	0.001627
50	0.219240	0.921782	0.700957	-0.020645	-0.023381	0.003779	-0.005127	1.406051	0.999912	0.006619
100	0.062262	0.361983	0.941185	0.044823	0.0570050	0.018037	0.013263	0.336943	0.999947	0.005130

6 Brush Heights, H

Table S9: Brush heights (r_c) for each grafting density (r_c^{-2}) and chain length. ν is the scaling exponent of the brush height with N: $H \sim N^{\nu}$. Uncertainties represent one standard deviation in the value.

σ (r_c^{-2})	$N = 20$	$N = 50$	$N = 100$	ν
0.25	6.87 ± 0.01	16.10 ± 0.04	31.78 ± 0.12	0.951
0.50	8.23 ± 0.01	17.63 ± 0.03	40.90 ± 0.05	0.988
0.75	9.36 ± 0.01	23.61 ± 0.01	47.30 ± 0.02	1.007
1.00	10.17 ± 0.01	25.91 ± 0.01	51.99 ± 0.03	1.014
1.25	10.82 ± 0.01	27.60 ± 0.01	55.74 ± 0.01	1.019
1.50	11.31 ± 0.01	28.96 ± 0.01	58.40 ± 0.03	1.020

7 Dynamical Anisotropy

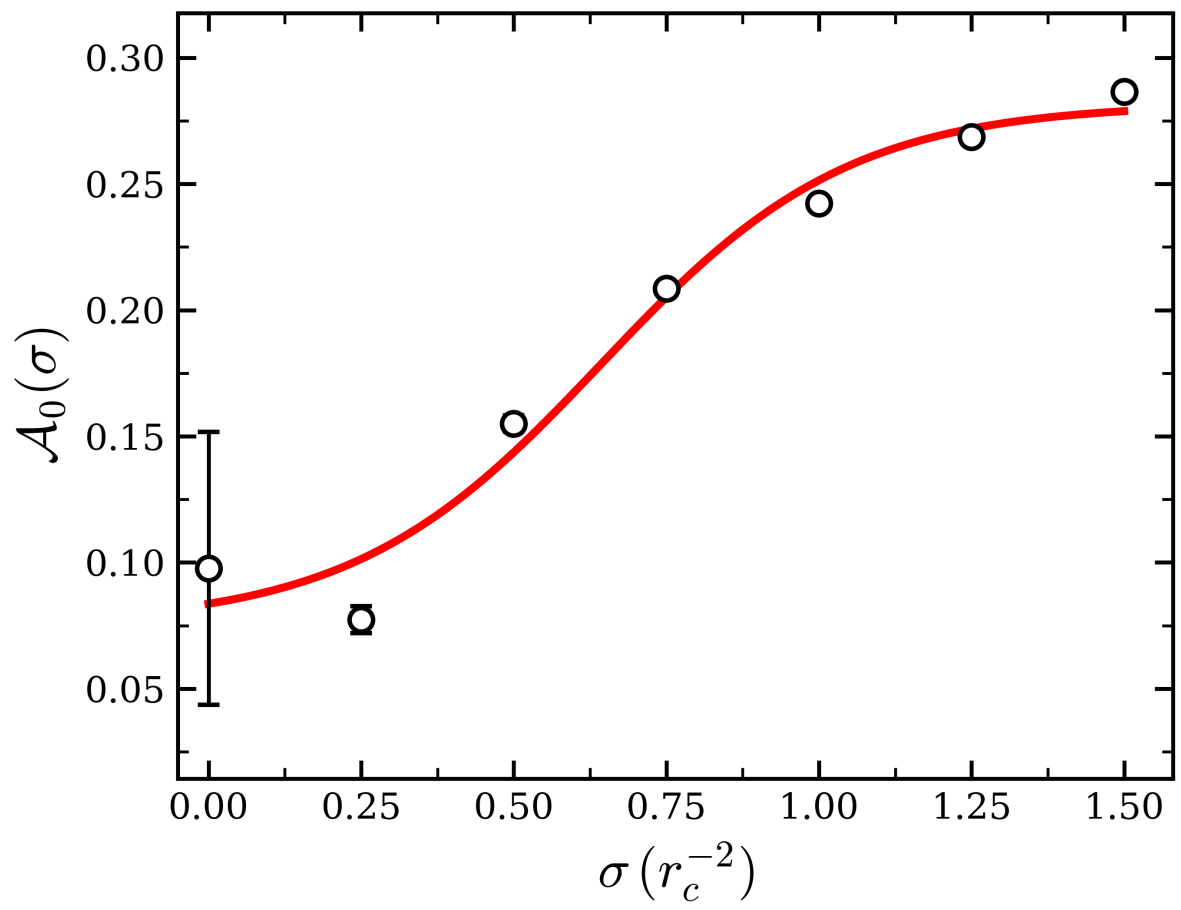

Figure S10: Contour-averaged dynamical anisotropy $\mathcal{A}_0$ as a function of grafting density σ for $N = 20$. Error bars represent one standard deviation in $\mathcal{A}_0$. The red line is a visual guide.

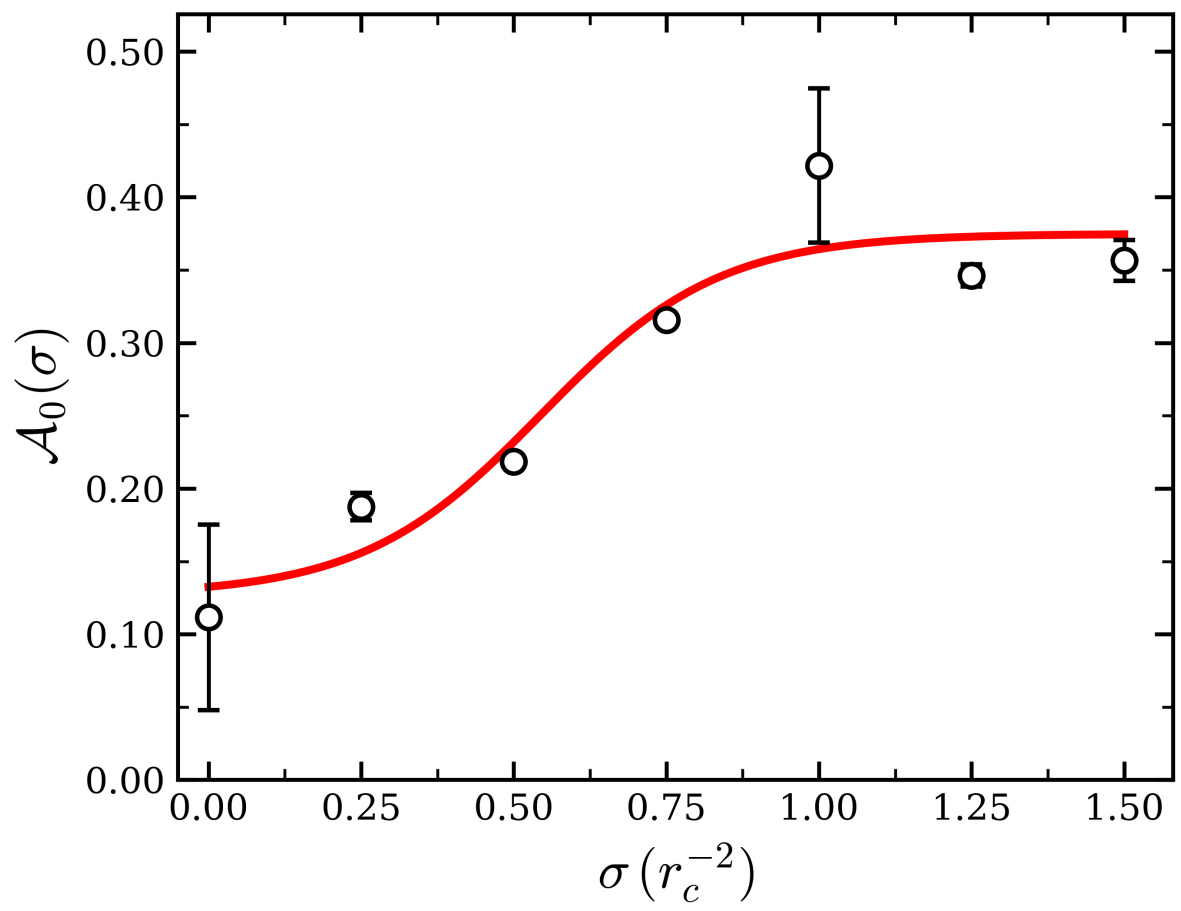

Figure S11: Contour-averaged dynamical anisotropy $\mathcal{A}_0$ as a function of grafting density σ for $N = 50$. Error bars represent one standard deviation in $\mathcal{A}_0$. The red line is a visual guide.

References

[1] Hore, M. J. A.; Laradji, M. Dissipative particle dynamics simulation of the interplay between spinodal decomposition and wetting in thin film binary fluids. *The Journal of Chemical Physics* **2010**, *132*.

[2] Hore, M. J. A. Analysis of the internal motions of thermoresponsive polymers and single chain nanoparticles. *Soft Matter* **2025**, *21*, 770–780.

[3] Lahmar, F.; Rousseau, B. Influence of the adjustable parameters of the DPD on the global and local dynamics of a polymer melt. *Polymer* **2007**, *48*, 3584–3592.

[4] Cheng, J.; Vishnyakov, A.; Neimark, A. V. Morphological transformations in polymer brushes in binary mixtures: DPD study. *Langmuir* **2014**, *30*, 12932–12940.

[5] Li, Z.; Tang, Y.-H.; Lei, H.; Caswell, B.; Karniadakis, G. E. Energy-conserving dissipative particle dynamics with temperature-dependent properties. *Journal of Computational Physics* **2014**, *265*, 113–127.

[6] Li, Z.; Tang, Y.-H.; Li, X.; Karniadakis, G. E. Mesoscale modeling of phase transition dynamics of thermoresponsive polymers. *Chemical Communications* **2015**, *51*, 11038–11040.

[7] Wong, C. P. J.; Choi, P. Analysis of Brownian dynamics and molecular dynamics data of unentangled polymer melts using proper orthogonal decomposition. *Macromolecular Theory and Simulations* **2019**, *28*, 1800072.

[8] Miller, C. A.; Hore, M. J. A. Simulation of the coronal dynamics of polymer-grafted nanoparticles. *ACS Polymers Au* **2021**, *2*, 157–168.

[9] Mukkamala, R. S.; Hore, M. J. A. Simulation and analysis of molecular bottlebrush dynamics in dilute solutions. *Macromolecules* **2024**, *57*, 445–455.

[10] Strobl, G. R. *The Physics of Polymers*, 2nd ed.; Springer: Berlin, 1997.
